# XIST Loss Induces Variable Transcriptional Responses Dependent on Cell States

**DOI:** 10.3390/ncrna11050067

**Published:** 2025-09-12

**Authors:** Dongning Chen, Ikrame Naciri, Jie Wu, Sha Sun

**Affiliations:** 1 Department of Developmental and Cell Biology, University of California, Irvine, CA 92697, USA; dongnic1@uci.edu (D.C.); inaciri@uci.edu (I.N.); 2 Department of Biological Chemistry and Genomics Research and Technology Hub (GRT Hub), University of California, Irvine, CA 92697, USA; jiew5@uci.edu

**Keywords:** XIST, X chromosome inactivation, dosage compensation

## Abstract

**Background/Objectives**: The X-inactivation specific transcript (XIST) is a long noncoding RNA playing a crucial regulatory role in X chromosome inactivation (XCI)—a transcriptional regulatory process that silences one of the two X chromosomes in females to ensure proper dosage compensation between male and female mammals. The transcription of *XIST* is maintained throughout a female’s lifespan in all somatic cells, where XIST RNA binds to the X chromosome in *cis* and ensures chromosome-wide gene silencing. Disrupting *XIST* expression can lead to transcriptional reactivation of X-linked genes and epigenetic changes affecting cell development. The prevalence of XIST regulatory effects on mammalian transcription, however, remains unclarified. **Methods**: Here we performed a comparative expression analysis using RNA-sequencing datasets from recently published studies and examined the consequences of XIST-deletion on transcription at the whole genome, individual chromosome, and specific gene levels. We investigated the common differentially expressed genes (DEGs) and biological pathways following XIST loss across cell types, together with differential transcriptional analysis comparing the X chromosome and autosomes using cumulative distribution fractions. We analyzed the distribution of DEGs along the X chromosome with scatterplots and correlation analysis incorporating gene density and transposable elements. **Results**: Our findings indicate that the loss of XIST causes transcriptional changes in the X chromosome and autosomes that differ depending on cell type and state. XIST-deletion results in differential expression of genes subject to XCI-silencing as well as genes escaping XCI. In all the cell types we analyzed, X-linked genes show differential expression across the entire X chromosome in a cluster-like pattern according to gene density and, in certain cell types, correlate strongly with short interspersed nuclear element (SINE) distributions. **Conclusions**: Our results demonstrate that transcriptional roles of XIST can be highly associated with cell state: stem cells have different transcriptional responses compared to differentiated cells following XIST loss.

## 1. Introduction

The human genome contains two sex chromosomes: females have two X chromosomes while males have one X chromosome and one Y chromosome. To resolve the X-linked gene dosage variation between the sexes, X chromosome inactivation (XCI) occurs starting at early embryonic development in females which silences the transcription of one of the two X chromosomes. The human long noncoding RNA XIST (X-inactivation specific transcript), homologous to mouse Xist, is required for initiating XCI and is exclusively expressed from the inactive X chromosome (Xi) [1,2,3,4]. During development, XCI is initially imprinted on the paternal X chromosome in mice during pre-implantation and becomes random post-implantation, whereas in humans, pre-implantation development is characterized by X chromosome dampening, with both X chromosomes being suppressed before transitioning to random XCI after implantation [4]. XCI propagates on the same Xi throughout cell cycles and silences genes on Xi. The transcription of *XIST* and the maintenance of XCI are crucial for maintaining female health and play significant roles in sex-biased diseases such as autoimmune diseases [5,6,7,8,9]. Moreover, abnormal *XIST* expression has been observed in various cancers, and studies have shown that transcription of *XIST* affects tumor progression [10,11,12,13,14].

As a paradigm for chromosomal epigenetic regulation and a model for RNA-protein interactions, XIST/Xist lncRNA-mediated XCI has been studied for decades using mouse Xist as the primary model. Xist activates XCI by binding in *cis* across the Xi, recruiting chromatin regulatory factors such as polycomb repressive complex (PRC1 and PRC2) to form the silent nuclear compartment [15,16,17]. During XCI initiation, Xist interacts with gene-rich regions based on spatial proximity, and the silent compartment formation alters the 3D chromosome architecture in a way that allows for more contact sites for Xist binding and promotes Xist spreading across the X chromosome to silence the entire Xi [18,19,20]. In parallel, *XIST*/*Xist* transcription and its lncRNA interactions with chromatin associated proteins can alter the structural conformation of the Xi; this results in loss of DNA accessibility and disruption of topologically associated domains, which contributes to X-linked gene silencing [21,22,23]. The heterochromatization of Xi has been observed with an enrichment of repressive epigenetic modifications, including histone 3 lysine 27 trimethylation (H3K27me3) and histone 3 lysine 9 trimethylation (H3K9me3), which are associated with transcriptional repression of X-linked genes [24,25]. However, there are species-specific differences: in mice, repressive histone marks are relatively uniform along the Xi, whereas in humans, the histone marks H3K27me3 and H3K9me3 show a spatially segregated pattern [26,27].

Although it has been shown that some mouse tissues can resist Xist deficiency, the loss of XIST/Xist upregulates X-linked genes, imbalances the gene dosage, alters cell functions, causes abnormal development, and induces pathological changes [13,28,29,30,31,32,33]. In human embryonic stem cells (ESCs), the loss of XIST triggers a phenomenon known as X-chromosome erosion, which is characterized by progressive DNA hypomethylation and ultimately irreversible X reactivation. Recent studies have also shown that this erosion results in a global increase in protein expression [34,35]. Importantly, this phenomenon appears to be species-specific, as erosion is not observed in mouse ESCs. Based on these differences, we hypothesize that XIST/Xist loss would have different effects depending on species and cell type, with stronger transcriptional impacts expected in undifferentiated cells compared to differentiated ones. To understand how XIST impacts XCI maintenance and gene regulation generally, we investigated the transcriptional response to XIST deficiency in various human and mouse cell lines, at the whole genome, individual chromosome, and specific gene levels. We used human ovarian cancer OVCAR3 cell line to generate XIST-knockdown cells and performed RNA-sequencing (RNA-seq) to determine the transcriptional changes associated with XIST deficiency [14]. Additional RNA-seq datasets were obtained from published studies of human mammary stem cells with XIST knockout and human embryonic stem cells with XIST deletion [13,36]. In parallel, we also analyzed published RNA-seq datasets from mouse embryonic fibroblasts and bone marrow cells with Xist deletion [33]. According to our comparative analysis, XIST deficiency results in significant transcriptional changes across all cell types, with limited overlap of differentially expressed genes (DEGs) between cell types, affecting a comparable proportion of genes on all chromosomes, not just X-linked genes. Different cell types show different tolerance to XIST deficiency and exhibit distinct transcriptional responses. In stem-like cells such as human embryonic stem cells and mouse hematopoietic stem cells, deleting XIST/Xist leads to significant upregulation of X-linked genes compared to autosomal genes and a change in chromosome dosage compensation. However, in differentiated cells such as human ovarian cancer cells and mouse embryonic fibroblasts, deletion of XIST/Xist affects all chromosomes similarly, and chromosome dosage compensation remains unchanged, suggesting that XIST/Xist is not necessary for XCI maintenance.

XIST deficiency caused differential expression of genes across the entire X chromosome, clustered according to gene density, in all cell types. We found that the density of transposable elements, long Interspersed nuclear element (LINEs), was highly correlated with the clustering of X-linked DEGs, particularly the upregulated ones, in embryonic stem cells with XIST-deletion; however, in differentiated cells, short interspersed nuclear elements (SINEs) were found to correlate more strongly with X-linked DEGs, especially upregulated genes. Additionally, we analyzed XCI inactive genes (genes that are subject to transcriptional silencing on Xi) and XCI escape genes (genes that escape transcriptional silencing on Xi) with allele-specific expression (ASE). In contrast to the reported significant reactivation of escape genes in mammary cells after XIST loss, ovarian cancer cells exhibit both XCI inactive and escape genes were equally affected by XIST loss. Overall, our comparative analysis highlights the importance of considering cell types and states when investigating transcriptional regulation associated with XIST/Xist function and its potential implications for development and disease.

## 2. Results

### 2.1. XIST/Xist Loss Induces Transcriptional Changes in the Human and Mouse Cells

**Figure 1:**
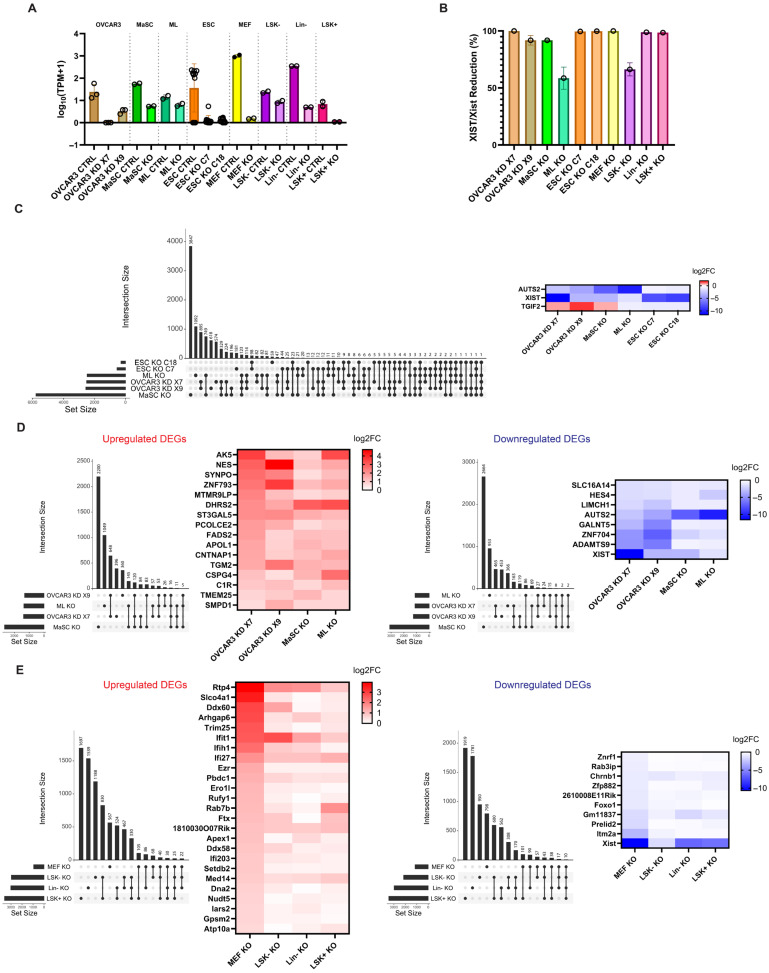
XIST loss induced cell type–specific transcriptional changes in human and mouse cells with limited overlap across cell types. (**A**) XIST expression levels (log_10_(TPM + 1)) in Control and XIST-deficient human and mouse cells. (**B**) Percentage reduction of XIST expression in XIST-deficient human and mouse cells compared to controls. (**C**) UpSet plot showing the overlap of DEGs following XIST loss across six human cell samples (OVCAR3 KD X7, OVCAR3 KD X9, MaSC KO, ML KO, ESC KO C7, ESC KO C18), alongside a heatmap displaying the corresponding log2FoldChange of DEGs shared in all six datasets. (**D**) UpSet plot showing the overlap of upregulated and downregulated DEGs following XIST loss among OVCAR3 (KD X7 and X9 clones), MaSC KO and ML KO cells, with heatmaps displaying log2FoldChange for each group. (**E**) UpSet plot showing the overlap of upregulated and downregulated DEGs following Xist loss among mouse cells (MEF KO, LSK− KO, Lin− KO and LSK+ KO), with heatmaps displaying their respective log2FoldChange. There are 3 biological replicates for OVCAR3 Control, KD X7 and KD X9 cells; 2 biological replicates for MaSC Control and KO, as well as ML Control and KO cells; 10 biological replicates for ESC Control and 11 biological replicates for ESC KO C7 and KO 18 clones; and 2 biological replicates for all mouse cell samples.

We characterized the expression levels of *XIST*/*Xist* across all cell types including control and XIST/Xist deficient samples (Figure 1A). Genetic deficiencies resulted in more than 90% reduction of *XIST*/*Xist* expression in almost all cell types, including human ovarian cancer cells (OVCAR3), human mammary stem cells (MaSC), human embryonic stem cells (ESC), mouse embryonic fibroblasts (MEF), mouse lineage-depleted (Lin−) bone marrow cells and the subgroup Lin^−^c-Kit^+^Sca-1^+^ (LSK+) cells; 50–70% reduction of *XIST*/*Xist* expression was resulted in human mammary luminal cells (ML) and mouse Lin^−^c-Kit^+^Sca-1^−^ (LSK−) cells (Figure 1B).

To understand the transcriptional effects of XIST loss in human cells, we investigated DEGs associated with XIST deficiency to identify genes consistently subject to XIST regulation across different cell types. As shown in Figure 1C, only two downregulated DEGs, *XIST* itself and *AUTS2*, were identified as overlapping across all cell types; no commonly upregulated DEG was found. Between human ovarian cancer cells and mammary cells, 16 upregulated and 8 downregulated DEGs were shared among the OVCAR3 KD X7 and X9, MaSC KO and ML KO samples; however, none of the shared DEGs were X-linked except for *XIST* itself (Figure 1D). For the transcriptional effects of Xist loss in mouse cells, 25 upregulated and 10 downregulated DEGs were identified as common across MEF KO, LSK− KO, Lin− KO and LSK+ KO cells, with the upregulated X-linked DEGs including *Arhgap6*, *Med14*, *Ftx* and *Pbdc1*, and a downregulated X-linked DEG, *Itm2a* (Figure 1E). There was no common DEG associated with XIST/Xist loss between human and mouse cells. Overall, our results suggest that XIST/Xist loss likely induces cell type specific transcriptional responses and that different genes would respond to XIST/Xist deficiency in human and mouse cells.

To understand whether XIST loss leads to alterations in similar biological pathways across different cell types, we performed a preranked Gene Set Enrichment analysis (GSEA) to identify significant enrichment of gene ontology biological process (GOBP) across multiple datasets. No common enriched GOBP terms were found across all six human cell datasets. In human ovarian cancer and mammary cells, we identified significant enrichment in four biological processes related to metabolism, cell morphogenesis and adhesion (Supplementary Figure S1A). However, these processes showed variable enrichment patterns: for example, cell junction assembly was positively enriched in OVCAR3 KD X7 and ML KO samples but negatively enriched in OVCAR3 KD X9 and MaSC KO samples. In mice, only one common pathway, response to type I interferon, was consistently enriched in hematopoietic stem and progenitor cells but not in MEFs (Supplementary Figure S1B). Thus, functional consequences of XIST/Xist loss depend on cell types.

### 2.2. The Loss of XIST in Human Cells Causes Variable Transcriptional Changes to the X Chromosome

To better understand the chromosomal effects of XIST loss, we analyzed transcriptomic data from various human cell lines. Since XIST is crucial for XCI, we hypothesized that XIST deletion would induce reactivation of transcriptionally silenced genes on the Xi and lead to upregulation of X-linked genes. Therefore, we investigated the upregulated differentially expressed genes (Up DEGs) associated with XIST loss and examined whether the X chromosome would exhibit a distinct transcriptional increase compared to other chromosomes. For upregulation of gene expression, we used a threshold of 1.5-fold (log2FoldChange > 0.58, padj < 0.05), considering the likely dose-dependent effect of XCI on Xi and Xa [37].

**Figure 2:**
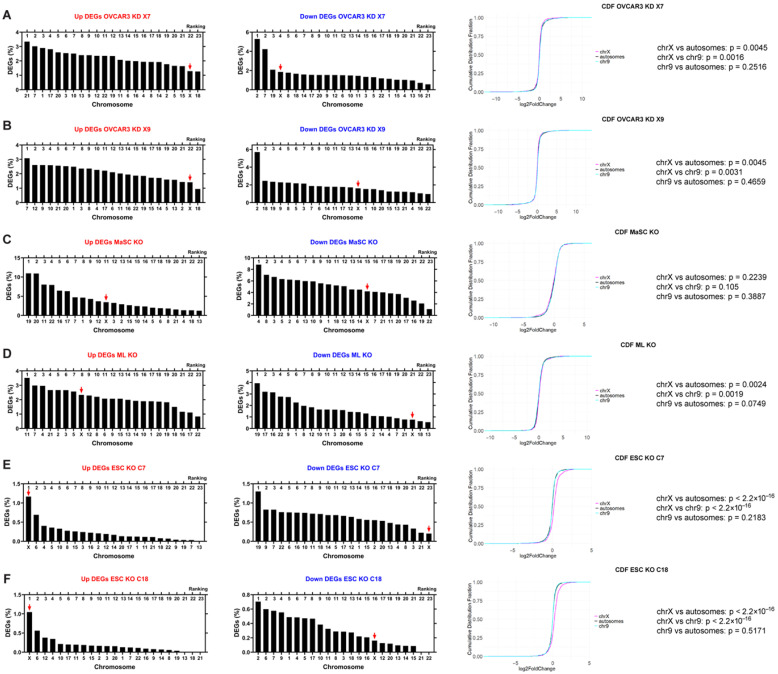
X chromosome exhibited different transcriptional changes compared to autosomes after XIST loss. The ranking of the percentages of upregulated DEGs (cutoffs by padj < 0.05 and log2FoldChange > 0.58) and downregulated DEGs (cutoffs by padj < 0.05 and log2FoldChange < −0.58) of each chromosome with X chromosome marked with a red arrow. The CDF plots of X chromosome, Chromosome 9 and autosomes with *p* values were calculated using a two-tailed Kolmogorov–Smirnov test. OVCAR3 KD X7 (**A**), OVCAR3 KD X9 (**B**), MaSC KO (**C**), ML KO (**D**), ESC KO C7 (**E**) and ESC KO C18 (**F**).

Contrary to our expectation of a higher representation of upregulated genes on the X chromosome with XIST deficiency, neither of the two XIST-knockdown OVCAR3 cell lines showed a significantly greater percentage of upregulated X-linked genes. In fact, across the whole genome, the X chromosome ranked 22nd out of 23 chromosomes for Up DEGs in both OVCAR3 KD X7 and KD X9 samples (Figure 2A,B). By contrast, the percentage of downregulated X-linked genes was higher than most other chromosomes, particularly in OVCAR3 KD X7 (ranked 4th, Figure 2A), and relatively lower in OVCAR3 KD X9 (ranked 14th, Figure 2B). From the cumulative distribution fraction (CDF) analysis, the X chromosome had a significantly left-shifted curve compared to autosomes or Chromosome 9, indicating transcriptional downregulation of the X compared to autosomes following XIST loss in ovarian cancer cells; no difference was observed when we compared Chromosome 9 with all autosomes (Figure 2A,B).

To determine whether similar effects would be observed in other cell types, we examined transcriptomic profiles of XIST knockout in human mammary stem cells (MaSC KO) and their differentiated progeny, mature luminal cells (ML KO), where XIST expression was inhibited [13]. Compared to OVCAR3 cells, a relatively higher percentage of X-linked genes were upregulated in these cells after XIST loss (the X chromosome ranked 11th and 8th out of 23 chromosomes, in MaSC KO and ML KO, respectively). By contrast, downregulated genes were less prevalent on the X chromosome (ranked 15th in MaSC KO and 21st in ML KO) (Figure 2C,D). The CDF curve confirmed that XIST loss led to significant upregulation of the X chromosome in comparison with autosomes in ML cells; no difference was observed when comparing Chromosome 9 with all autosomes (Figure 2D).

In addition, we examined the effect of XIST loss on human pluripotent stem cells by analyzing transcriptomic data from two human embryonic stem cell (hESC) clones, ESC KO C7 and ESC KO C18, in which XIST was deleted [36]. In both clonal cell lines with loss of XIST, the X chromosome ranked 1st among all chromosomes for upregulated genes (Figure 2E,F) and was among the least affected chromosomes for downregulated genes, ranking 23rd for ESC KO C7 cells and 16th for ESC KO C18 cells (Figure 2E,F). The CDF curves further confirmed significant upregulation of the X chromosome compared to all autosomes and Chromosome 9; no significant difference was observed between Chromosome 9 and all autosomes (Figure 2E,F).

When we performed a Z test comparing the percentage of DEGs on each chromosome, we found no significant difference between the X chromosome and autosomes, whether for all DEGs, upregulated DEGs, or downregulated DEGs that resulted from XIST loss, in all cells we analyzed (Supplementary Table S1). Moreover, there was no significant difference in the percentage of DEGs between the top-ranked chromosome and the other chromosomes (Supplementary Table S1), indicating that DEG proportions are similar across chromosomes, and that the deletion of XIST affects the transcription of all chromosomes nearly equally. Together, our analysis shows that XIST loss is associated with transcriptional changes of the X chromosome and autosomes in human cells, and distinct upregulation of X-linked genes is only observed in human embryonic stem cells but not in lineage-specific or cancer cells.

### 2.3. The Loss of Xist in Mouse Cells Causes Variable Transcriptional Changes to the X Chromosome

**Figure 3:**
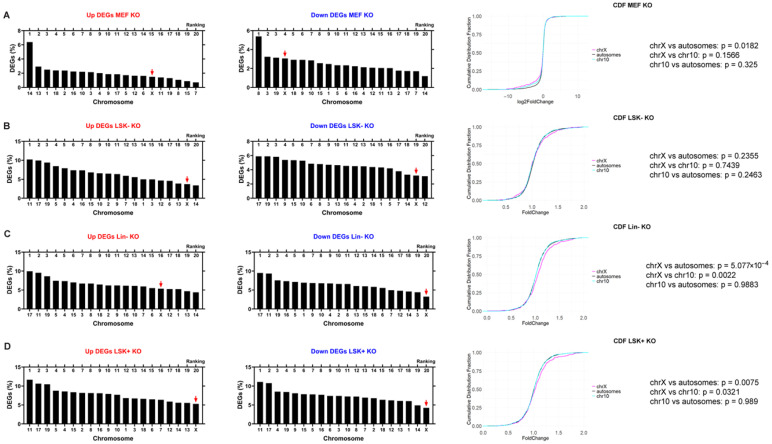
Xist knockout led to distinct regulations in X chromosome transcription in mouse cells. The ranking of the percentages of upregulated DEGs and downregulated DEGs of each chromosome with X chromosome marked with a red arrow. The DEGs dataset of MEF were generated by cutoffs of padj < 0.05 & log2FoldChange > 0.58 for upregulated DEGs and padj < 0.05 & log2FoldChange < −0.58 for downregulated DEGs. The DEGs datasets of LSK−, Lin− and LSK+ were extracted from Supplementary Data from the Yildirim lab [33]. The CDF plots of X chromosome, Chromosome 10 and autosomes with *p* values were calculated using a two-tailed Kolmogorov–Smirnov test. MEF KO (**A**), LSK− KO (**B**), Lin− KO (**C**), and LSK+ KO (**D**).

To determine whether Xist loss affects transcription of the X chromosome in mouse cells similarly to human cells, we analyzed the transcriptomic data from differentiated mouse embryonic fibroblasts (MEFs) with Xist deletion [33]. The X chromosome ranked 15th out of 20 chromosomes according to the percentage of upregulated DEGs, whereas it ranked 4th out of 20 for downregulated DEGs (Figure 3A). In agreement with the human data, Supplementary Table S1 indicates that the loss of Xist affects the percentage of DEGs across each chromosome comparably in all mouse cells. The CDF curves showed a significantly left-shifted curve for the X chromosome when compared to autosomes, indicating that Xist loss in MEFs led to significant downregulation of the X; there was no significant difference between Chromosome 10 and all autosomes (Figure 3A).

To examine the effect of Xist loss in less differentiated cell states, we used transcriptomic data generated by the Yildirim group from studying mouse hematopoietic cells with Xist deletion: Lin-cells contained both pluripotent and multipotent cells of the hematopoietic lineage, which were further divided into LSK+ cells (mainly pluripotent hematopoietic stem cells) and LSK− cells (more differentiated, multipotent cells committed to the myeloid and lymphoid lineage) [33]. In these three cell lines with Xist loss, the X chromosome appeared to be one of the least affected chromosomes: the X ranked 16th, 19th, 20th for upregulated DEGs and 20th, 19th, 20th for downregulated DEGs (Figure 3B–D). The CDF curves for LSK− KO cells showed no significant difference in X chromosome transcription compared to autosomes or Chromosome 10 (Figure 3B). In contrast, the CDF curves for Lin− KO cells, which contain both LSK+ KO and LSK− KO populations revealed a significant right-shift for the X chromosome compared to autosomes or Chromosome 10 (Figure 3C), indicating upregulation of the X. The CDF curves for LSK+ KO cells, the most stem-like among these three cell lines, showed a significant right-shift for the X chromosome compared to autosomes or Chromosome 10 (Figure 3D). These results are consistent with the Yildirim lab’s findings which indicate that dosage compensation defects following Xist loss accumulate as cells undergo differentiation [33]. In general, the X chromosome is susceptible to Xist loss in varying degrees, as in human cells, and transcriptional upregulation of the mouse X chromosome also depends on the cell state.

### 2.4. XIST Loss Causes Transcriptional Changes of XCI Inactive Genes and XCI Escape Genes in Ovarian Cancer Cells

**Figure 4:**
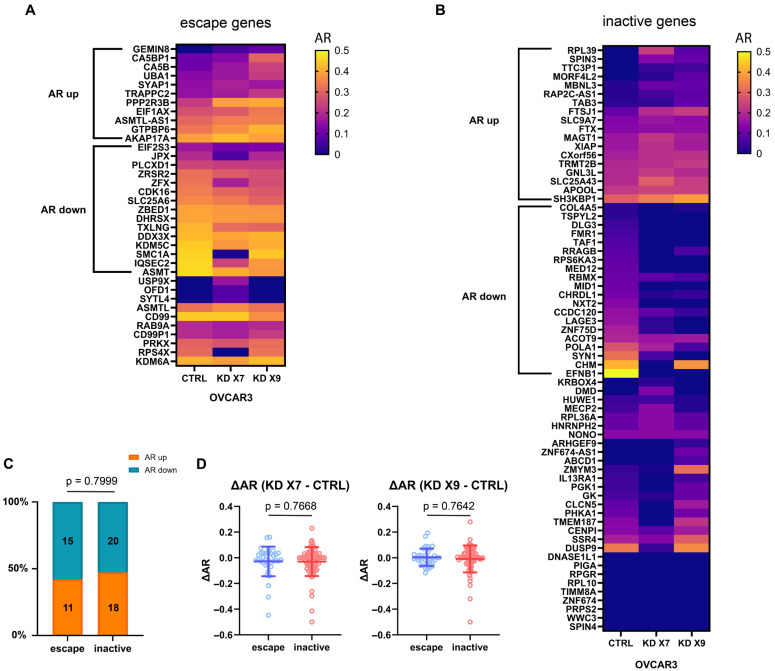
XIST knockdown alters allelic expression of both escape and inactive genes equally in OVCAR3. (**A**,**B**) Heatmaps showing the allelic ratio (AR) calculated as the ratio of reads mapping to the minor allele over the total reads (AR = min[refCount, altCount]/(refCount + altCount)) of escape and inactive genes across OVCAR3 CTRL and two XIST knockdown clones (KD X7 and KD X9). Genes were classified based on previously reported XCI status as listed (Supplementary Excel File S1). AR = 0 means full monoallelic and AR = 0.5 means full biallelic expression. Subsets labelled with AR up or down were genes with increased or decreased AR in both knockdown clones. (**C**) Bar plot showing the contingency table comparing the proportion of escape or inactive genes that exhibited increased or decreased AR in both KD X7 and KD X9 clones. Statistical significance was tested by Fisher’s exact test. (**D**) Scatter plots comparing the ΔAR (KD—CTRL) between escape and inactive genes for OVCAR3 KD X7 (left) and KD X9 (right). Each dot represented an individual gene. Normal distribution of the data was assessed using the Shapiro–Wilk test, and statistical significance was tested using the Mann–Whitney U test.

The transcriptional effects of XCI can be leaky, allowing genes to escape silencing: 15–25% of human X-linked genes escape XCI compared with 3–7% in mice [38,39]. Compared to its effects on XCI inactive genes, it has been shown that XIST loss tends to reactivate XCI escape genes—particularly those located on the X short arm and within Polycomb-repressed domains [13]. Additional studies with human induced pluripotent stem cells (hiPSCs) demonstrated that XIST loss primarily reactivates XCI escape genes within the X chromosome pseudoautosomal region 1 (PAR1) [40,41]. We hypothesized that XIST loss might preferentially reactivate escape genes by upregulating their expression on the Xi. To investigate how XIST deficiency impacts gene expression across XCI status (inactive or escape), we performed allele-specific expression (ASE) analysis in OVCAR3 cells comparing control (CTRL) with two XIST knockdown clones (KD X7 and KD X9). After XIST knockdown, 11 escape genes, including *PPP2R3B* and *GTPBP6*, and 18 inactive genes, including *FTSJ1* and *SH3KBP1*, showed increased allelic ratio (AR) in both clones, suggesting enhanced biallelic expression in these subsets of genes (Figure 4A,B). Additionally, there were 15 escape and 20 inactive genes displayed decreased AR in both XIST knockdown clones (Figure 4A,B), indicating enhanced monoallelic expression. The proportion of escape genes with increased AR was comparable to that of inactive genes (Figure 4C), suggesting that XIST loss impacts X-linked allelic expression regardless of XCI status. Furthermore, there was no significant difference in the alteration of AR (ΔAR) for escape genes and inactive genes (Figure 4D), suggesting that XIST loss induces comparable effects on transcription for escape genes and inactive genes.

### 2.5. XIST/Xist Loss Leads to Transcriptional Changes in Genes Clustered Along the X Chromosome

**Figure 5:**
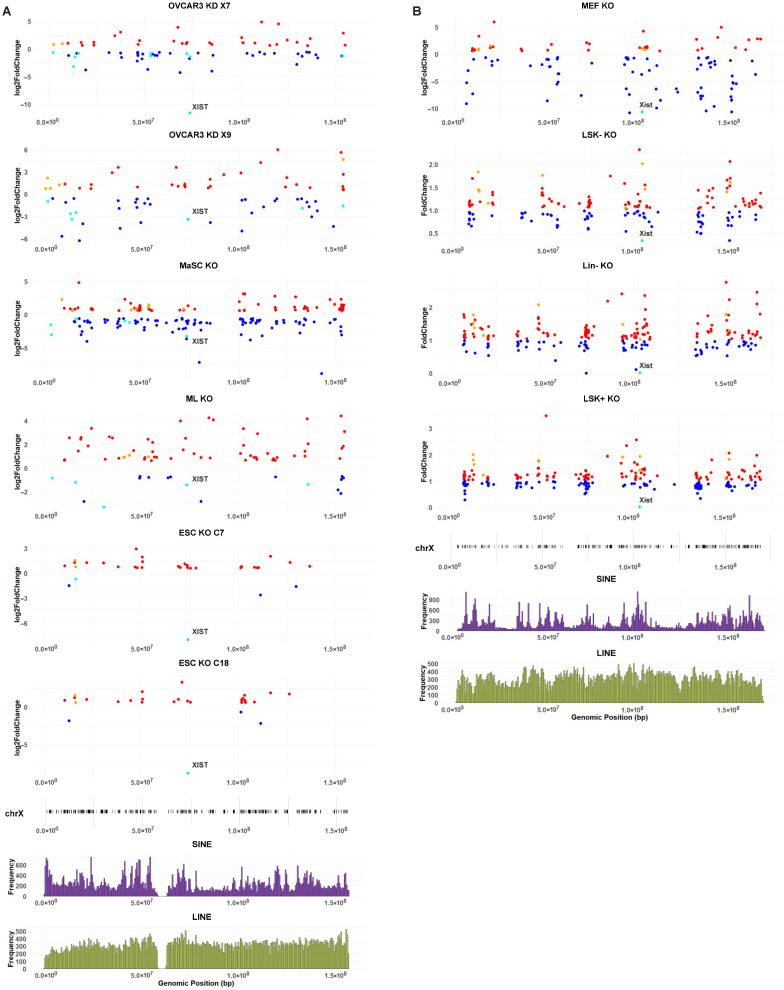
X-linked DEGs due to XIST loss were distributed along the X chromosome forming multiple clusters driven by gene density. The scatterplots of DEGs on the X Chromosome according to their genomic positions: (**A**) human cells: OVCAR3 KD X7, OVCAR3 KD X9, MaSC KO, ML KO, ESC KO C7 and ESC KO C18; (**B**) mouse cells: MEF KO, LSK− KO, Lin− KO and LSK+ KO. The x-axis was a genomic position calculated by the mean of start and end bp of each DEG. The y-axis was the log2FoldChange of each DEG analyzed by DESeq2 with cutoffs of padj < 0.05 and |log2FoldChange| > 0.58. The upregulated DEGs with log2FoldChange > 0.58 were in red with XCI escape genes in orange and the downregulated DEGs with log2FoldChange < −0.58 were in blue with XCI escape genes in cyan; XIST was labeled and marked in green. Following the scatterplots of DEGs, gene density, SINEs and LINEs along the X chromosome were plotted respectively.

We investigated how DEGs associated with XIST/Xist loss are distributed across the X chromosome according to their genomic positions. In human cells with XIST deficiency, the X-linked DEGs (all, upregulated, and downregulated) were distributed in clusters throughout the entire X (Figure 5A). A positive correlation between the density of X-linked DEGs and all X-linked genes suggests that the cluster-like pattern of DEG distribution is associated with gene density (Supplementary Table S2). The correlation strength varied across cell types with a low correlation in hESC (KO C7 clone: R = 0.2960 and KO C18 clone: R = 0.3294) compared to a high correlation in MaSC KO cells (R = 0.5737). The correlation of X-linked DEGs between different clonal lines of the same human cells, such as ESC KO C7 and ESC KO C18 (R = 0.6568), was stronger than comparing different cell types, such as OVCAR3 KD X7 and ML KO cells (R = 0.2538); there was no significant correlation between ESC KO C7 and ML KO cells (Supplementary Table S3). The outcomes suggest that transcriptional changes associated with XIST loss are dependent on cell types.

In addition, we found that X-linked DEGs associated with XIST loss were correlated with the density of transposable elements, LINEs but not SINES, in human ESCs; however, in human ovarian cancer and mammary cells, X-linked DEGs showed a higher correlation with SINEs than LINEs (Figure 5A; Supplementary Figure S2; Supplementary Table S4). Given that SINE are predominantly enriched in gene-rich regions [42,43], we performed a partial correlation analysis between X-linked DEGs and SINE density when controlling for gene density. In human ovarian cancer cells, the correlation between X-linked DEGs and SINE density remained significantly positive after controlling for gene density, indicating a likely association between SINEs and transcriptional changes caused by XIST loss independently of gene-rich regions. However, in human mammary cells, the correlation between X-linked DEGs and SINE density was largely diminished after adjusting for gene density, suggesting that in these cells, the correlation distribution of transcriptional changes caused by XIST loss was primarily attributable to gene density rather than a specific association with SINEs (Supplementary Table S5).

When examining the upregulated DEGs and downregulated DEGs, separately, in human ovarian cancer and mammary cells with XIST loss, it was observed that both up- and down-regulated DEGs were better associated with SINEs than LINEs, except in MaSC KO cells, where downregulated DEGs were more closely associated with LINEs than SINEs (Supplementary Table S6). After controlling for gene density, upregulated DEGs in OVCAR3 KD X7 and downregulated DEGs in OVCAR3 KD X9 remained significantly and positively in correlation with SINEs (Supplementary Table S7). By contrast, upregulated DEGs in human ESCs with XIST loss correlated more strongly with LINEs (Figure 5B; Supplementary Table S6).

In parallel, we analyzed mouse X-linked gene expression affected by Xist loss. We plotted the distribution of DEGs along the X chromosome according to genomic positions (Figure 5B); the scatterplots confirmed that DEGs (all, upregulated and downregulated) were distributed across the entire X chromosome. Consistent with observations in the human data, the distribution showed a cluster-like pattern associated with gene density, as evidenced by a positive correlation between X-linked DEGs and all genes on the X chromosome (Supplementary Table S2). There were significantly positive correlations for X-linked DEGs between different mouse cell lines with Xist loss: high correlations were observed among hematopoietic stem and progenitor cells (R = 0.8579 between Lin− KO and LSK+ KO; R = 0.8610 between Lin− KO and LSK− KO). In comparison, the correlation for X-linked DEGs between hematopoietic cells and embryonic fibroblasts was lower (R = 0.3016 between Lin− KO and MEF KO). As observed in human cells, X-linked DEGs resulting from Xist loss in mouse cells were clustered according to gene density, and cell state affects X-linked transcriptional changes associated with Xist loss (Supplementary Table S3).

In all mouse cells with Xist deficiency, X-linked DEGs were strongly correlated with SINE density (Supplementary Table S4). This positive correlation remained statistically significant after accounting for gene density, suggesting a direct association between SINEs and X-linked transcriptional changes following XIST loss (Supplementary Table S5). In the hematopoietic cell lineages with Xist loss, both upregulated and downregulated X-linked DEGs were positively correlated with SINEs while negatively correlated with LINEs (Supplementary Table S6). In MEF cells with Xist loss, both upregulated and downregulated X-linked DEGs were positively correlated with SINEs but not correlated with LINEs. The differential correlations with SINEs and LINEs could be explained by an anti-correlation between SINE and LINE densities along the mouse X chromosome (R = −0.1628, *p* = 1.89 × 10^−5^; Supplementary Figure S3). When controlling for gene density, the significant positive correlation with SINEs persisted for both upregulated and downregulated DEGs in Lin− and LSK− cells; but in LSK+ and MEF, only the upregulated DEGs retained a significant correlation with SINEs (Supplementary Table S7).

Taken together, the cluster-like distribution of X-linked DEGs caused by XIST/Xist loss is commonly correlated with gene density in human and mouse cells. In addition, the loss of XIST in human ESCs affects transcription of X-linked genes associated mainly with LINEs, and Xist loss in mouse cells affects transcription of X-linked genes associated with SINEs.

## 3. Discussion

In this study, we compared the transcriptional effects of XIST/Xist loss across different cell lines derived from humans and mice. Due to differences in experimental conditions, we did not merge raw RNA-seq data. We analyzed differential signatures resulting from XIST loss across different cell states. Based on differential gene expression, we found that X-linked genes were more strongly affected by XIST/Xist in undifferentiated cell states, both in humans and mice. At the same time, our analysis revealed that both human and mouse cells exhibit distinct transcriptional responses to XIST/Xist loss. Notably, XIST/Xist loss does not lead to massive reactivation of the X chromosome compared to autosomes, except in early embryonic cells. Our observations are consistent with XIST/Xist’s critical function during early development. Indeed, it is well established that XCI is initiated in the early stages of development in both humans and mice [44]. Upregulation of the X chromosome in human ESCs and mouse hematopoietic stem cells with XIST/Xist deletion is consistent with XCI defects after XIST/Xist loss. In well-differentiated cells such as human cancer cells and mouse fibroblasts, XIST/Xist deletion leads to downregulation of the X chromosome compared to autosomes, suggesting XCI-independent transcriptional changes after XIST/Xist loss.

In human MaSCs, XIST loss did not result in X chromosome reactivation compared to autosomes, but in progenitor ML cells, XIST loss led to significant X chromosome reactivation. A possible explanation is that ML cells derived from MaSCs may accumulate transcriptional changes during differentiation, resulting in more pronounced reactivation of X-linked genes in ML cells. This possibility is supported by published work from the Yildirim lab, which shows a similar accumulation of XCI defects in Lin− KO cells composed of progenitor and pluripotent cells in mice. Importantly, when comparing the XIST/Xist expression levels across different cell types, we observed that the transcriptional changes of the X chromosome compared to autosomes, whether upregulation or downregulation, were not dependent on *XIST*/*Xist* expression levels or the efficiency of XIST/Xist reduction (Supplementary Table S8), suggesting a dose-independent effect of XIST/Xist on X chromosome regulation.

The differential impact of XIST/Xist loss across cell types can be explained by its developmental expression patterns and the underlying epigenetic state of the inactive X chromosome. In ESCs, where XIST/Xist is required to initiate and stabilize X-chromosome inactivation within a permissive chromatin environment, its loss results in widespread transcriptional reactivation of X-linked genes [45,46]. In contrast, in differentiated cells, silencing becomes progressively stabilized by additional layers of repression, including promoter DNA methylation and repressive histone modifications such as H3K27me3, rendering the Xi largely independent of continued XIST/Xist expression for maintenance [32]. This locked epigenetic state explains why XIST/Xist depletion in differentiated cells has comparatively modest effects. Cancer cells, however, represent a distinct case: many exhibit features of X chromosome erosion, including reduced XIST/Xist expression, focal loss of H3K27me3, and unstable DNA methylation [47]. In this context, XIST/Xist loss may produce heterogeneous outcomes, ranging from partial reactivation to selective downregulation of X-linked genes, depending on lineage-specific and tumor-specific chromatin alterations.

We have focused on the chromosome-wide transcriptional changes associated with XIST/Xist loss via analyzing the percentages of DEGs on each chromosome. In general, our analysis shows that only a small subset of X-linked genes, which make up 1–4% for the human X and 1–6% for the mouse X (Supplementary Excel File S2), are reactivated after XIST/Xist deletion. This suggests that there are potential compensatory mechanisms to maintain XCI, mitigate adverse effects, and preserve normal cellular states and functions. This finding is further supported by published evidence that: female mice lacking Xist show only modest overexpression of X-linked genes and persists partial dosage compensation [48], and the deletion of Xist in the brain results in reactivation in only 2–5% of neurons [30]. Recent studies have also shown that XIST/Xist may directly affect autosomal genes [36,49,50]. While we hypothesized that the X chromosome is more susceptible to transcriptional reactivation caused by XIST deficiency, we found that only in ESC does the X chromosome rank 1st in upregulated DEGs. This pattern aligns with the CDF plots and further supports the finding that transcriptional reactivation of the X chromosome compared to autosomes following XIST loss is dependent on developmental and cell status.

When examining the effect of XCI status on allelic expression changes following XIST knockdown in OVCAR3, we observed that XIST loss results in comparable transcriptional changes across escape genes and inactive genes rather than preferentially affecting escape genes. This observation contrasts with previous reports that escape genes are more susceptible to transcriptional reactivation upon XIST/Xist deletion in human mammary cells and mouse hemopoietic cells [13,33]. XIST loss in cancer cells may be subject to an altered epigenetic profile that modifies transcriptional control of X-linked genes. Additionally, the tissue- and cell-specific nature of XIST-mediated XCI has been documented [38,51], supporting the heterogeneous sensitivity to XIST loss across various cell types.

In all human datasets we analyzed, CRISPR-based techniques were employed to knockdown or knockout XIST in OVCAR3 cells, mammary cells, and human ESCs. Our OVCAR3 study used a CRISPR-Cas9 system with guide RNA targeting the XIST promoter to achieve transcriptional repression of XIST [14]. Similarly, a CRISPR-Cas9 cassette was inserted with guide RNA targeting XIST exon 1, effectively disrupting XIST transcription in MaSC and ML cells [13]. Additionally, the Plath group generated XIST knockout ESCs by removing the first 2kb of XIST exon 1 [36]. Despite different CRISPR strategies, the resulting DEGs were not clustered around the genomic region of guide-RNA targeting sites. Instead, they were distributed across the entire X chromosome, forming clusters according to gene density, a pattern consistent across all XIST-deficient cells (Figure 5). These results indicate that XIST exerts a global regulatory role on X-linked gene expression, regardless of the specific CRISPR technique employed.

We recognize that transposable elements such as LINEs and SINEs may play distinct roles in modulating XCI. LINEs are reported to facilitate XIST spreading and silencing across the X chromosome and are mainly enriched in the XCI region [52,53,54,55]. SINEs are generally less enriched on the X chromosome but more prevalent in XCI escape genes [54,55]. Importantly, the upregulated X-linked DEGs in human ESCs were correlated with LINEs, suggesting a reactivation of XCI silenced genes after XIST loss. However, this pattern was not observed in differentiated human cells. By contrast, SINEs and X-lined DEGs are strongly correlated in all mouse cells, especially for upregulated DEGs, likely due to the prevalence of SINEs in XCI escape genes predominately affected by Xist loss in mice. Our findings support that developmental and cell state could be a critical factor in transcriptional changes associated with XCI defects and XIST/Xist loss in different species.

In summary, XIST/Xist deficiency triggers variable genome-wide impacts in modulating the transcription of both the X chromosome and autosomes while the pattern of differential gene expression varies across different human/mouse cell types. The differential impact of XIST/Xist deficiency on X-linked genes underscores the complexity and heterogeneity of XCI and its regulation. Our findings contribute to a deeper understanding of the role of XIST/Xist in gene expression regulation and the evolutionary complexity of XCI.

## 4. Materials and Methods

### 4.1. RNA-Seq Datasets of Human and Mouse Cells with XIST/Xist Loss

We generated OVCAR3 RNA-seq profiles including the XIST knockdown cell lines OVCAR3 KD X7 and OVCAR3 KD X9 with the control OVCAR3 CTRL (GEO: GSE271117) [14]. CRISPRi with two guide RNAs targeting the XIST promoter (sgXIST7 5′GCAGCGCTTTAAGAACTGAA 3′; sgXIST9 5′GCCATATTTCTTACTCTCTCG 3′) and a control guide RNA targeting the Gal4 promoter (sgCtl: 5′ GAACGACTAGTTAGGCGTGT 3′) was used to knockdown XIST expression in the human ovarian cancer OVCAR3 cells. We obtained RNA-seq data of XIST knockout in human mammary stem cell (MaSC) and mammary luminal cell (ML) as published by the Ginestier group (accessed through GEO: GSE159946) [13], and RNA-seq data of XIST knockout in human embryonic stem cell (ESC) as published by the Plath group (accessed through GEO: GSE241444) [36]. In human mammary epithelial cells, XIST knockout was achieved by CRISPR-mediated insertion of a stop cassette into XIST exon 1 (5′- GCAGGTATCCG ATACCCCGA-3′), and sorting of MaSC and ML subpopulations. In human ESCs, XIST knockout was performed by CRISPR-mediated excising the first ~2kb of XIST using two guide RNA targeting the XIST promoter (chrX:73852766-73852785; hg38) and exon 1 (chrX:73850789-73850808; hg38), resulting in the generation of two XIST-knockout clonal lines, ESC KO C7 and ESC KO C18. For our comparative characterization, the counts data were normalized by DESeq2 for DEG analysis.

In parallel, we included RNA-seq data of Xist knockout in mouse hematopoietic stem cells and progenitor cells (HSPCs) within lineage-depleted (Lin−) bone marrow cells as published by the Yildirim group [33]. The Xist knockout derived DEGs dataset of Lin− cells, and its two subgroups Lin^−^c-Kit^+^Sca-1^+^ (LSK+) and Lin^−^c-Kit^+^Sca-1^−^ (LSK−) cells were downloaded from the published Supplementary Data [33]. The aligned read data of mouse embryonic fibroblasts (MEF) generated using TopHat2 were shared with us by the Yildirim group. The mouse Xist knockout cells, including MEF KO and the three HSPC subtypes (LSK− KO, Lin− KO and LSK+ KO), were isolated from Xist conditional knockout mice. FeatureCounts was used to count the aligned reads, and DESeq2 was used to normalize the count data for the DEG analysis.

For comparison of specific gene expression across datasets, such as XIST expression levels, we used transcript per million (TPM) normalization, which adjusts for gene length and sequencing depth. For differential expression analysis, we performed DESeq2 independently in each dataset and applied a uniform DEG cutoff for comparative analysis.

### 4.2. Transcriptional Effect Analysis and Statistics

To compare the *XIST* expression levels in control (with wild-type XIST; WT) and XIST-deficient cells, raw count data were transformed into transcript per million (TPM), and the percentage reduction of *XIST* expression in XIST-deficient cells relative to WT controls was calculated. Upregulated DEGs were determined by cutoffs of padj < 0.05 and log2FoldChange > 0.58 (FoldChange > 1.5) and downregulated DEGs were determined by cutoffs of padj < 0.05 and log2FoldChange < −0.58 (FoldChange < 0.67). For mouse HSPCs (Lin−, LSK+ and LSK−), DEG list was extracted from the published Supplementary Data by the Yildirim group [33]. Venn diagrams illustrating the overlap of upregulated and downregulated DEGs were generated using R, and heatmaps displaying log2FoldChange were plotted using GraphPad Prism 10.

To identify biological pathways commonly affected by XIST loss across multiple cell types, the preranked GSEA was performed using the clusterProfiler R package (4.12.6) [56] based on the GOBP database as the reference. For all human cells and MEFs, genes were ranked by the signed Wald statistic (stat) from DESeq2 (1.44.0). Significantly enriched GOBP terms were identified based on *p* adjust less than 0.05. For mouse hematopoietic stem and progenitor cells, enriched terms were available in the published Supplementary Data [33].

The percentages of DEGs were calculated by dividing the number of DEGs of each chromosome by the total number of genes on that chromosome and ranked and plotted as a histogram in descending order using GraphPad Prism 10. The comparison of the percentage of DEGs of chromosome X and the average percentage of autosomes was analyzed using the Z test; the comparison of the percentage of DEGs of the top-ranked chromosome and the average percentage of DEGs of the remaining chromosomes was analyzed using the Z test.

To compare the transcriptional changes to the X chromosome, Chromosome 9 (which has a gene count closest to that of the X chromosome in humans), and all autosomes in humans, log2FoldChange data of each human cell type analyzed by DESeq2 were used to plot the cumulative distribution fraction (CDF) graph of the X chromosome, Chromosome 9, and all autosomes. To compare the transcriptional changes to the X chromosome, Chromosome 10 (which has a gene count closest to that of the X chromosome in mice), and all autosomes in mice, FoldChange data of all actively expressed genes (FPKM ≥ 1) in Lin− KO, LSK+ KO and LSK− KO and log2FoldChange data of MEF KO generated by DESeq2 were used to plot the CDF graph. The statistical significance was analyzed using a two-tailed Kolmogorov–Smirnov test.

To evaluate the effect of XIST loss in the allelic expression of escape and inactive genes in OVCAR3, the heterozygous single nucleotide polymorphisms (SNPs) were called and counted from RNA-seq data across all OVCAR3 samples using GATK RNA best practices (with minimum read count cutoff of 10). SNPs were annotated to genes based on the hg38 genome. The allelic ratio (AR) was calculated as the ratio of reads mapping to the minor allele over the total reads (AR = min[refCount, altCount]/(refCount + altCount)). Genes with informative AR were then classified by their XCI status using previously published data (the combined XCI status published in Tukiainen et al. 2017 which integrated XCI data from Cotton et al. 2013 and Carrel & Willard 2005) [38,57,58]. The complete list of genes with AR values and their annotated XCI status is provided in Supplementary Excel File S1.

To examine the distribution of DEGs along the X chromosome, X-linked DEGs from the OVCAR3 KD, MaSC KO, ML KO, ESC KO and MEF KO datasets generated by DESeq2 were selected by cutoffs of padj < 0.05 and |log2FoldChange| > 0.58 and these DEGs were then used for scatter plots. Their log2FoldChanges were plotted according to their genomic positions along the X chromosome. The genomic positions were calculated by averaging the start and end base pairs (bp) of each gene. Upregulated (log2FoldChange > 0.58) and downregulated (log2FoldChange < −0.58) DEGs were marked in red and blue respectively, and XIST was labeled. To generate scatterplots of Lin− KO, LSK+ KO and LSK− KO cells, DEGs were extracted from the published Supplementary Data by the Yildirim group, and FoldChange was calculated by dividing Xist Δ/Δ FRKM by WT FPKM and followed by averaging the results from two replicates. Upregulated (FoldChange > 1) and downregulated (FoldChange < 1) DEGs were marked in red and blue respectively, and Xist was labeled. Pearson correlations between the enrichment densities of X-linked DEGs and either all genes, SINEs or LINEs on the X chromosome (using 1 Mbp window size and 250 kbp step size) were analyzed in RStudio (2024.12.1) using cor.test function to calculate *p* value and correlation coefficient (R). The window size and step size were as described [36,53]. The partial correlations between X-linked DEGs and SINEs in control of gene density were analyzed using ppcor (1.1) package in RStudio.

## Data Availability

The bulk RNA-seq data of human XIST-knockdown OVCAR3 generated by our lab are available on GSE271117 [14]. The published bulk RNA-seq data of human XIST knockout mammary cells and embryonic stem cells are downloaded from GSE159946 and GSE241444 respectively [13,36]. The published bulk RNA-seq data of mouse Xist knockout lineage-depleted (Lin−) bone marrow cells, LSK+ (Lin^−^c-Kit^+^Sca-1^+^) and LSK− (Lin^−^c-Kit^+^Sca-1^−^) cells are downloaded from the published Supplementary Data [33]. The AR data with XCI status in allelic expression analysis are available in the Supplementary Excel File S1. The percentages of DEGs of each chromosome and their rankings are available in the Supplementary Excel File S2. All codes for genomic analyses are available at https://github.com/DongningChen/ChrEffectXISTLoss (accessed on 28 April 2025).

## Supplementary Materials

The following supporting information can be downloaded at: https://www.mdpi.com/article/10.3390/ncrna11050067/s1, Supplementary Figure S1: (A) GOBP terms commonly enriched in human ovarian and mammary cells; (B) GOBP terms commonly enriched in mouse hematopoietic stem and progenitor cells. Supplementary Figure S2: Correlation between X-linked DEGs and LINE/SINE elements across XIST/Xist deficient human and mouse cell models. Supplementary Table S1: Statistical comparison between the percentage of DEGs of a specific chromosome and the remaining chromosomes. Supplementary Table S2: The Pearson correlation between DEG distribution and gene density on X chromosome in human and mouse cells. Supplementary Figure S3: Line plot of SINE and LINE densities along the X chromosome. Supplementary Table S3: The Pearson correlation of DEG distribution on X chromosome between different cell types. Supplementary Table S4: The Pearson correlation between SINE/LINE densities and X-linked DEGs in different human and mouse cells. Supplementary Table S5: The partial Pearson correlation between SINE densities and X-linked DEGs with control of gene density in human and mouse cells. Supplementary Table S6: The Pearson correlation between SINE/LINE densities and upregulated or downregulated X-linked DEGs in human and mouse cells. Supplementary Table S7: The Pearson correlation between SINE densities and upregulated or downregulated X-linked DEGs with control of gene density in human and mouse cells. Supplementary Table S8: The XIST expression level (TPM) and the transcription alteration of X chromosome compared to autosomes. Supplementary Excel File S1: The AR data with XCI status in OVCAR3. Supplementary Excel File S2: The percentages of DEGs of each chromosome and their rankings in all analyzed cells.
