# Supplementary material for: XIST Loss Induces Variable Transcriptional Responses Dependent on Cell States
Source: Noncoding RNA. 2025 Sep 12;11(5):67. doi: 10.3390/ncrna11050067 (PMC12452299; doi:10.3390/ncrna11050067)
Supplement: Supplementary file 1 [file ncrna-11-00067-s001.zip › Supplementary/Supplementary Tables S1 to S8.pdf]

Supplementary Table S1. Statistical comparison between the percentage of DEGs of a specific chromosome and the remaining chromosomes

		comparison between X chromosome and autosomes	comparison between the top ranked chromosome and the other chromosomes
	**Dataset**	**p value**	**p value**
**Human cells**	OVCAR3 KD X7 all DEGs	0.9695	0.8951
	OVCAR3 KD X7 up DEGs	0.9484	0.938
	OVCAR3 KD X7 down DEGs	0.988	0.7716
	OVCAR3 KD X9 all DEGs	0.9731	0.9471
	OVCAR3 KD X9 up DEGs	0.9616	0.9428
	OVCAR3 KD X9 down DEGs	0.9841	0.7725
	MaSC KO all DEGs	0.9988	0.8216
	MaSC KO up DEGs	0.9704	0.7299
	MaSC KO down DEGs	0.9739	0.8513
	ML KO all DEGs	0.9744	0.9414
	ML KO up DEGs	0.9886	0.922
	ML KO down DEGs	0.9406	0.8608
	ESC KO C7 all DEGs	0.99	0.9529
	ESC KO C7 up DEGs	0.8434	0.8434
	ESC KO C7 down DEGs	0.9561	0.9303
	ESC KO C18 all DEGs	0.9386	0.9161
	ESC KO C18 up DEGs	0.8435	0.8435
	ESC KO C18 down DEGs	0.9788	0.941
**Mouse cells**	LSK+ KO all DEGs	0.8742	0.8246
	LSK+ KO up DEGs	0.926	0.8781
	LSK+ KO down DEGs	0.9029	0.8831
	LSK- KO all DEGs	0.8919	0.8701
	LSK- KO up DEGs	0.9098	0.8738
	LSK- KO down DEGs	0.9438	0.9504
	Lin- KO all DEGs	0.8959	0.8407
	Lin- KO up DEGs	0.9608	0.8882
	Lin- KO down DEGs	0.8966	0.8937
	MEF KO all DEGs	0.9871	0.9222
	MEF KO up DEGs	0.9683	0.7517
	MEF KO down DEGs	0.9714	0.8498

Supplementary Table S2. The Pearson correlation between DEG distribution and gene density on X chromosome in human and mouse cells

**Human Cells**			**Mouse Cells**		
	**R**	**p value**		**R**	**p value**
**OVCAR3 KD X7**	0.4316	< 2.2e-16	**Lin- KO**	0.7197	< 2.2e-16
**OVCAR3 KD X9**	0.4785	< 2.2e-16	**LSK+ KO**	0.7902	< 2.2e-16
**MaSC KO**	0.5737	< 2.2e-16	**LSK- KO**	0.7794	< 2.2e-16
**ML KO**	0.4748	< 2.2e-16	**MEF KO**	0.3994	< 2.2e-16
**ESC KO C7**	0.2960	< 2.2e-16			
**ESC KO C18**	0.3294	< 2.2e-16			

Supplementary Table S3. The Pearson correlation of DEG distribution on X chromosome between different cell types

**Human Cells**			
		**R**	**p value**
**OVCAR3 KD X7**	**OVCAR3 KD X9**	0.6559	< 2.2e-16
**MaSC KO**	**ML KO**	0.6030	< 2.2e-16
**ESC KO C7**	**ESC KO C18**	0.6568	< 2.2e-16
**OVCAR3 KD X7**	**MaSC KO**	0.3740	< 2.2e-16
**OVCAR3 KD X7**	**ML KO**	0.2538	< 2.2e-16
**OVCAR3 KD X7**	**ESC KO C7**	0.3587	< 2.2e-16
**MaSC KO**	**ESC KO C7**	0.2780	3.52e-11
**ML KO**	**ESC KO C7**	0.0245	0.5669
**Mouse Cells**			
**Lin- KO**	**LSK+ KO**	0.8579	< 2.2e-16
**Lin- KO**	**LSK- KO**	0.8610	< 2.2e-16
**Lin- KO**	**MEF KO**	0.3016	< 2.2e-16

Supplementary Table S4. The Pearson correlation between SINE/LINE densities and X-linked DEGs in different human and mouse cells

Human cells				Mouse cells			
		R	p value			R	p value
**OVCAR3 KD X7**	SINE	0.3597	<2.2e-16	**Lin- KO**	SINE	0.7470	<2.2e-16
	LINE	0.1295	0.0012		LINE	-0.1639	1.81e-5
**OVCAR3 KD X9**	SINE	0.3801	<2.2e-16	**LSK+ KO**	SINE	0.6592	<2.2e-16
	LINE	0.0527	0.1911		LINE	-0.1845	1.32e-6
**MaSC KO**	SINE	0.2844	6.03e-13	**LSK- KO**	SINE	0.6973	<2.2e-16
	LINE	0.1647	3.96e-5		LINE	-0.1398	0.0003
**ML KO**	SINE	0.1832	4.57e-6	**MEF KO**	SINE	0.3546	<2.2e-16
	LINE	0.1442	0.0003		LINE	0.0103	0.7883
**ESC KO C7**	SINE	0.0658	0.0897	**All X-linked genes**	SINE	0.5781	<2.2e-16
	LINE	0.1612	0.0002		LINE	-0.1177	0.0021
**ESC KO C18**	SINE	0.0526	0.2377				
	LINE	0.1172	0.0084				
**All X-linked genes**	SINE	0.4937	<2.2e-16				
	LINE	0.2401	1.21e-9				

Supplementary Table S5. The partial Pearson correlation between SINE densities and X-linked DEGs with control of gene density in human and mouse cells

Human Cells			Mouse Cells		
	**R**	**p value**		**R**	**p value**
**OVCAR3 KD X7**	0.1852	3.44e-6	**Lin- KO**	0.5952	< 2.2e-16
**OVCAR3 KD X9**	0.1813	5.97e-6	**LSK+ KO**	0.4239	< 2.2e-16
**MaSC KO**	-0.0165	0.6820	**LSK- KO**	0.4982	< 2.2e-16
**ML KO**	-0.0788	0.0504	**MEF KO**	0.1859	1.11e-6
**ESC KO C7**	-0.0549	0.1996			
**ESC KO C18**	-0.1779	5.91e-5			

Supplementary Table S6. The Pearson correlation between SINE/LINE densities and upregulated or downregulated X-linked DEGs in human and mouse cells

**Human cells**				
			**R**	**p**
**OVCAR3 KD X7**	Up DEG	SINE	0.2978	3.49e-14
		LINE	0.1199	0.0028
	Down DEG	SINE	0.2375	2.39e-9
		LINE	0.0839	0.0375
**OVCAR3 KD X9**	Up DEG	SINE	0.2395	1.71e-9
		LINE	0.0302	0.4541
	Down DEG	SINE	0.3410	<2.2e-16
		LINE	0.0517	0.2001
**MaSC KO**	Up DEG	SINE	0.3124	1.96e-15
		LINE	0.1027	0.0107
	Down DEG	SINE	0.0829	0.0402
		LINE	0.1582	8.35e-5
**ML KO**	Up DEG	SINE	0.1525	0.0001
		LINE	0.1548	0.0001
	Down DEG	SINE	0.1336	0.0009
		LINE	0.0507	0.2084
**ESC KO C7**	Up DEG	SINE	0.1274	0.0028
		LINE	0.1872	1.02e-5
	Down DEG	SINE	0.0515	0.2413
		LINE	0.0067	0.8783
**ESC KO C18**	Up DEG	SINE	0.0436	0.3277
		LINE	0.1147	0.0099
	Down DEG	SINE	0.0513	0.2800
		LINE	0.0513	0.2801

**Mouse cells**				
**Lin- KO**	Up DEG	SINE	0.7047	<2.2e-16
		LINE	-0.1740	5.16e-6
	Down DEG	SINE	0.6115	<2.2e-16
		LINE	-0.1097	0.0046
**LSK+ KO**	Up DEG	SINE	0.7426	<2.2e-16
		LINE	-0.2287	1.70e-9
	Down DEG	SINE	0.4383	<2.2e-16
		LINE	-0.1067	0.0058
**LSK- KO**	Up DEG	SINE	0.6544	<2.2e-16
		LINE	-0.1511	7.82e-5
	Down DEG	SINE	0.4947	<2.2e-16
		LINE	-0.0825	0.0331
**MEF KO**	Up DEG	SINE	0.3700	<2.2e-16
		LINE	-0.1096	0.0043
	Down DEG	SINE	0.2051	9.36e-8
		LINE	0.0658	0.0897

Supplementary Table S7. The Pearson correlation between SINE densities and upregulated or downregulated X-linked DEGs with control of gene density in human and mouse cells

**Human cells**				
			**R**	**p**
**OVCAR3 KD X7**	Up DEG	SINE	0.1708	1.90e-5
	Down DEG	SINE	0.0708	0.0793
**OVCAR3 KD X9**	Up DEG	SINE	0.0690	0.0869
	Down DEG	SINE	0.1924	1.54e-6
**MaSC KO**	Up DEG	SINE	0.0121	0.7640
	Down DEG	SINE	-0.0641	0.1131
**ML KO**	Up DEG	SINE	-0.0134	0.7402
	Down DEG	SINE	-0.1500	0.0002
**ESC KO C7**	Up DEG	SINE	-0.0643	0.1330
	Down DEG	SINE	0.0058	0.8949
**ESC KO C18**	Up DEG	SINE	-0.1886	2.02e-5
	Down DEG	SINE	-0.1050	0.8258
**Mouse cells**				
**Lin- KO**	Up DEG	SINE	0.5118	<2.2e-16
	Down DEG	SINE	0.3604	<2.2e-16
**LSK+ KO**	Up DEG	SINE	0.5549	<2.2e-16
	Down DEG	SINE	-0.0037	0.9251
**LSK- KO**	Up DEG	SINE	0.4568	<2.2e-16
	Down DEG	SINE	0.1078	0.0054
**MEF KO**	Up DEG	SINE	0.1958	2.83e-7
	Down DEG	SINE	0.0650	0.0938

Supplementary Table S8. The XIST expression level (TPM) and the transcription alteration of X chromosome compared to autosomes

Cell Type	XIST expression (TPM)	XIST reduction (%)	Transcriptional regulation of X compared to autosomes
OVCAR3 KD X7	0.01004	99.9651	Downregulation
OVCAR3 KD X9	2.31574	91.9408	Downregulation
MaSC KO	4.47853	91.8270	No difference
ML KO	5.40668	58.5842	Upregulation
ESC KO C7	0.52208	99.5730	Upregulation
ESC KO C18	0.30137	99.7535	Upregulation
Lin- KO	3.97788	99.9456	Upregulation
LSK+ KO	0.09099	66.2831	Upregulation
LSK- KO	7.67812	98.8618	No difference
MEF KO	0.53799	98.5190	Downregulation
