# Supplementary figures and images for: XIST Loss Induces Variable Transcriptional Responses Dependent on Cell States
Source: Noncoding RNA. 2025 Sep 12;11(5):67. doi: 10.3390/ncrna11050067 (PMC12452299; doi:10.3390/ncrna11050067)

Supplementary Figure S1

A

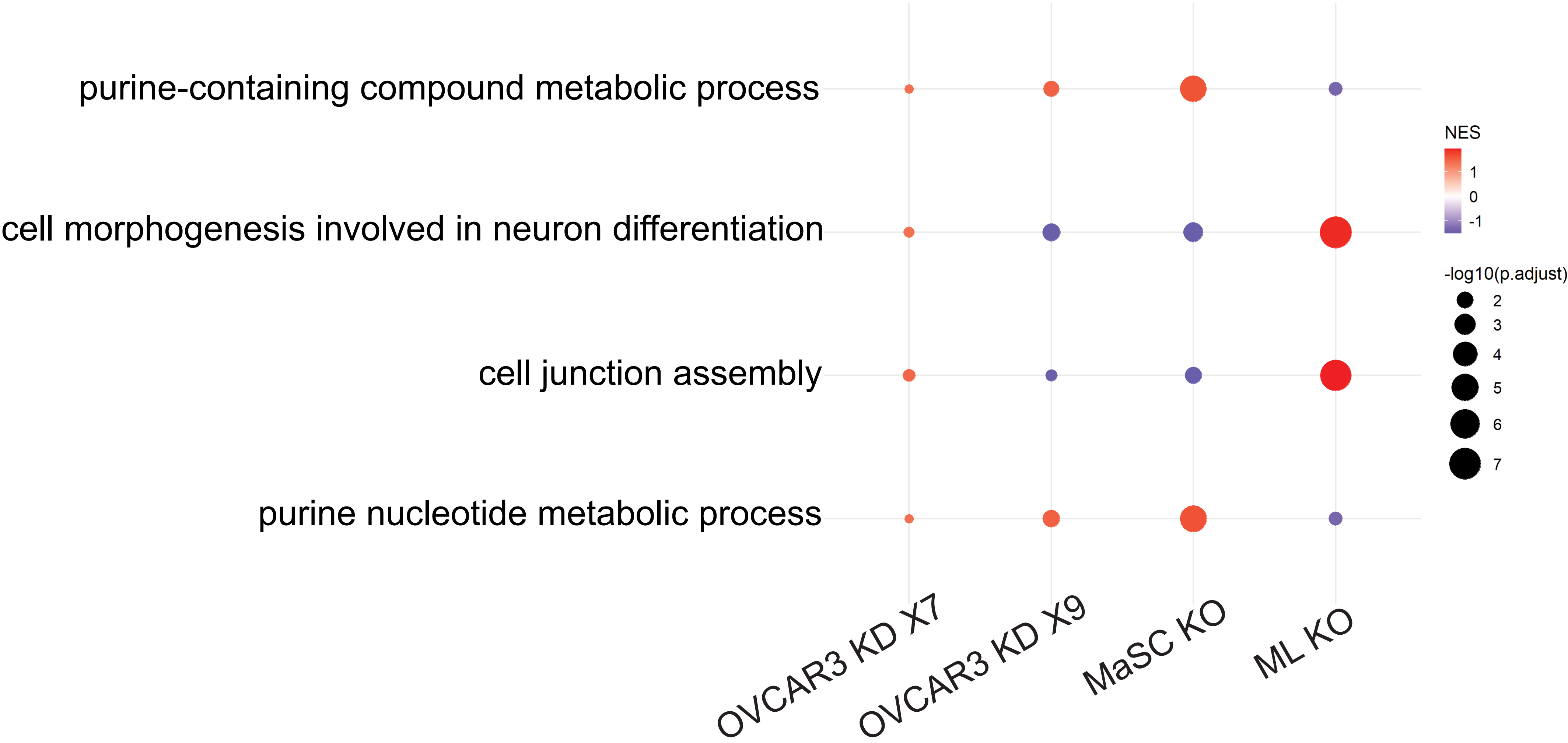

B

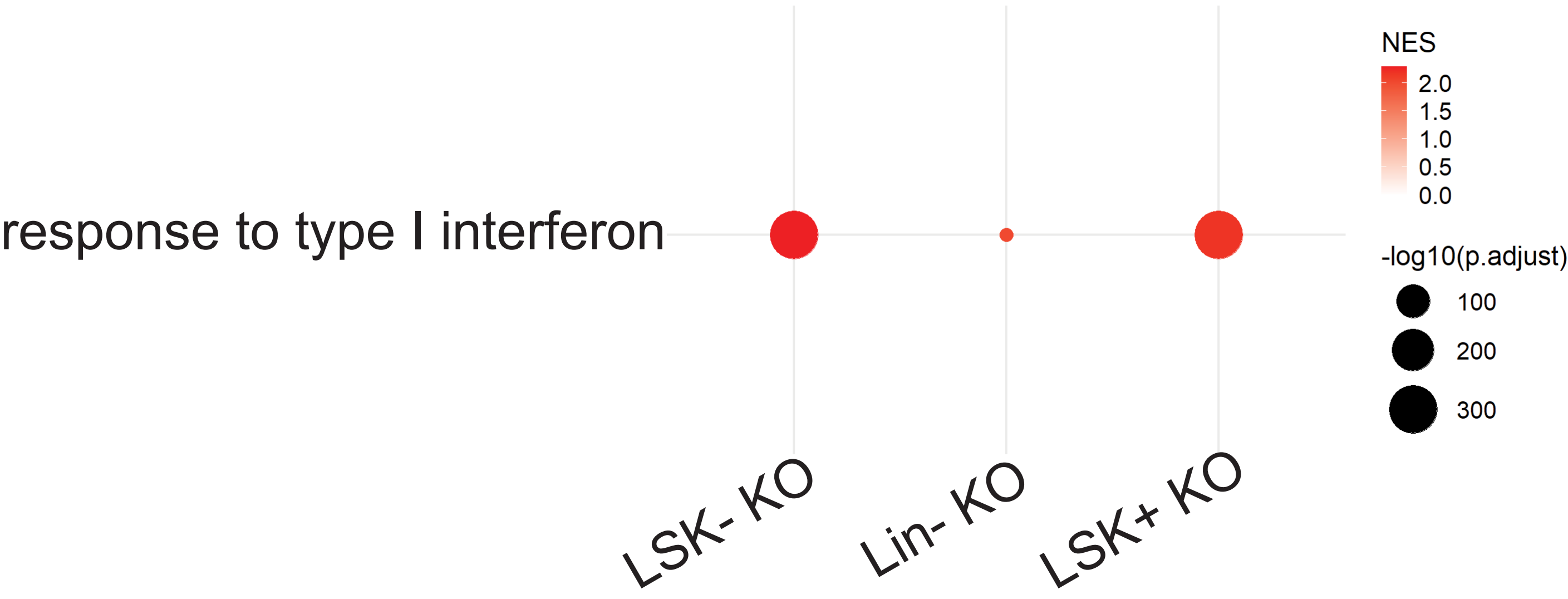

Supplementary Figure S2

A

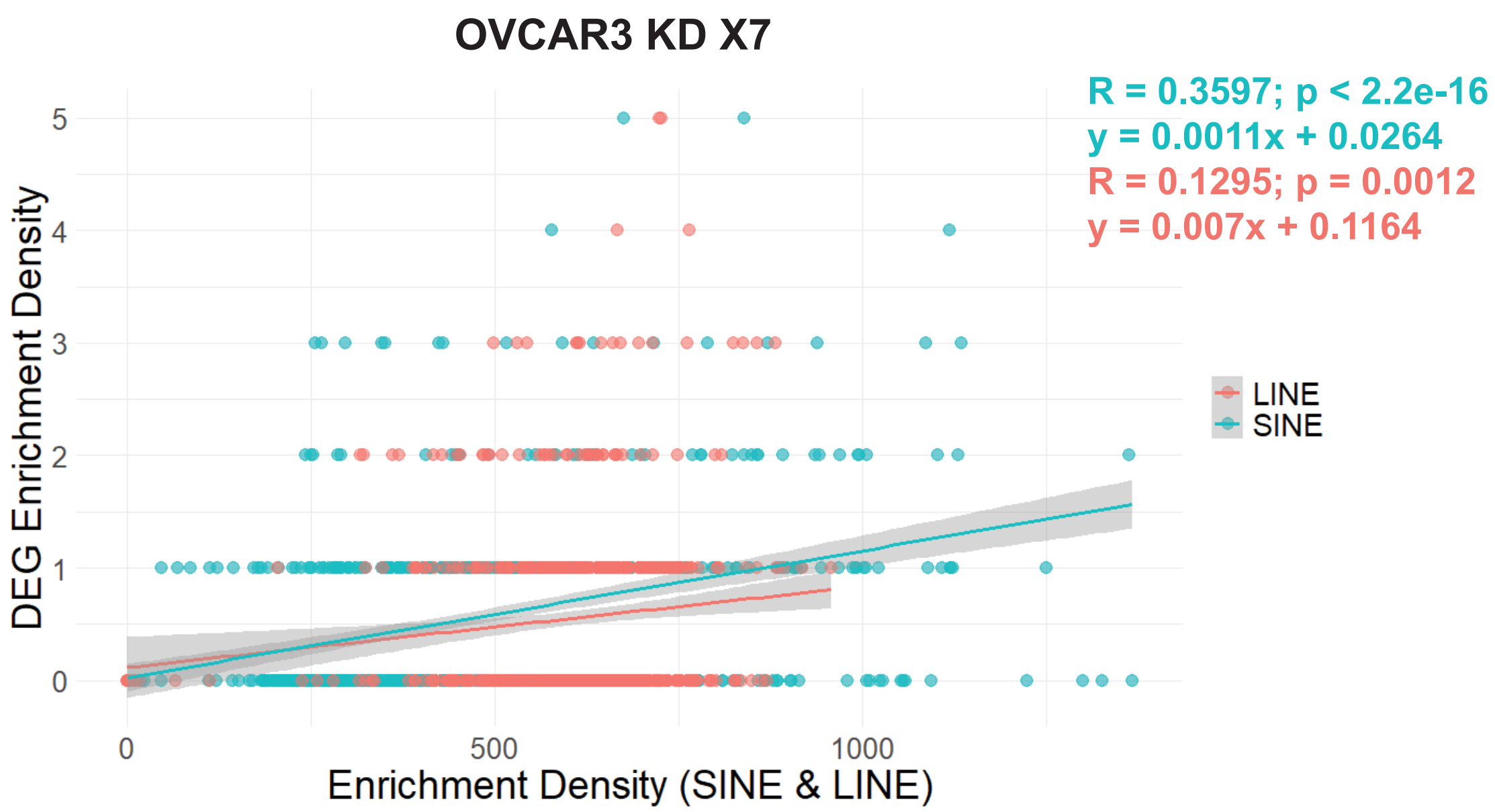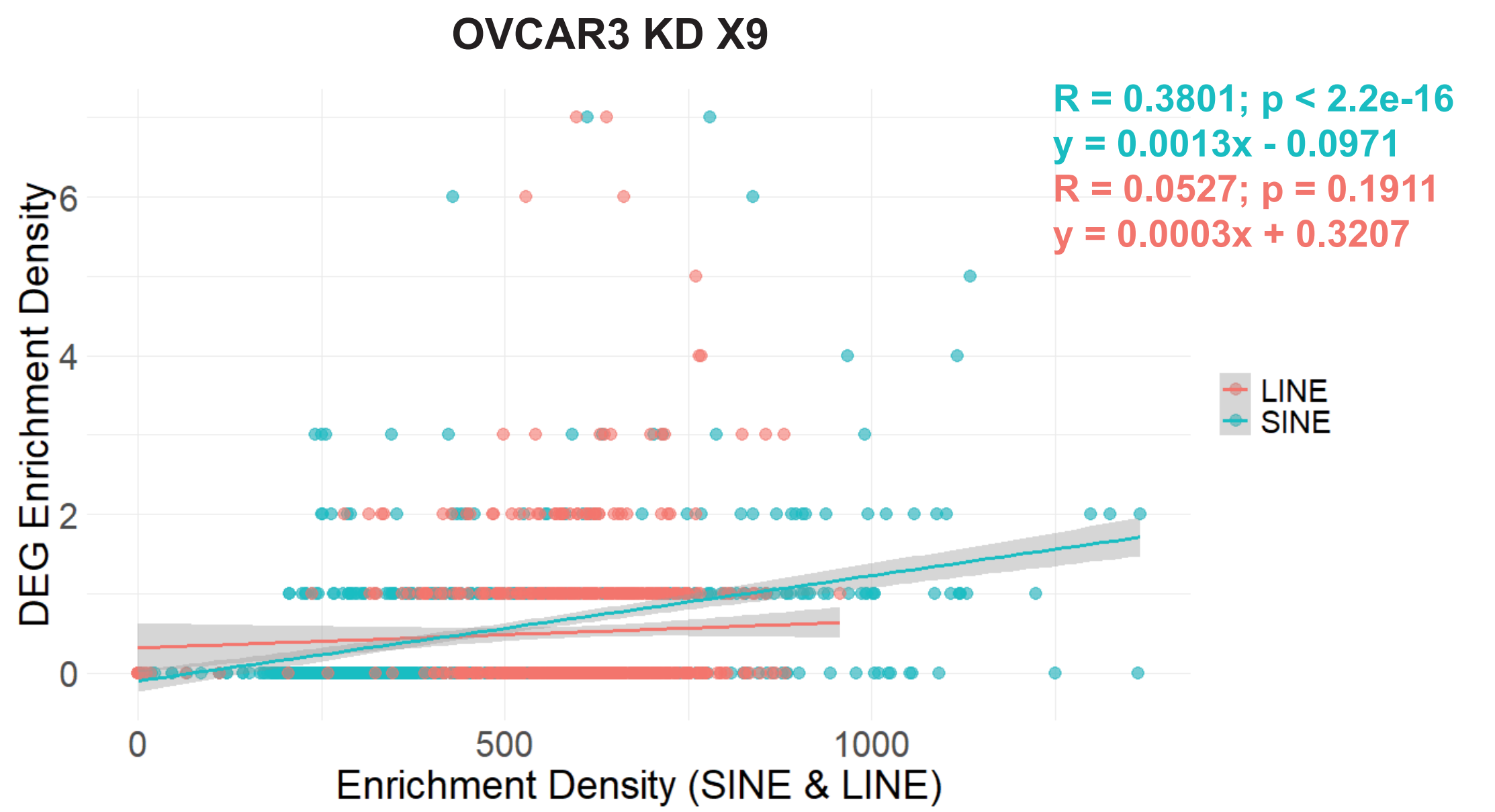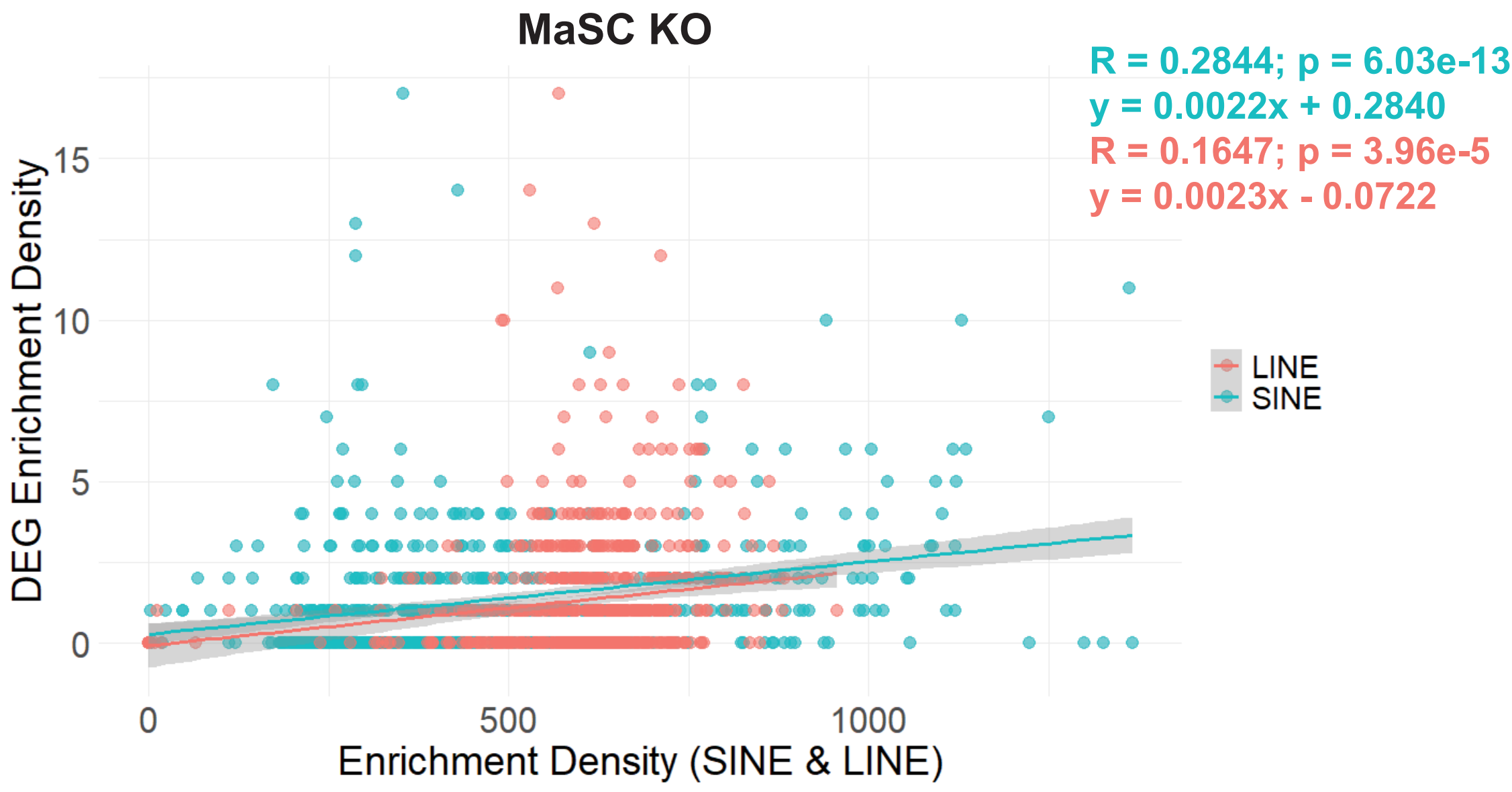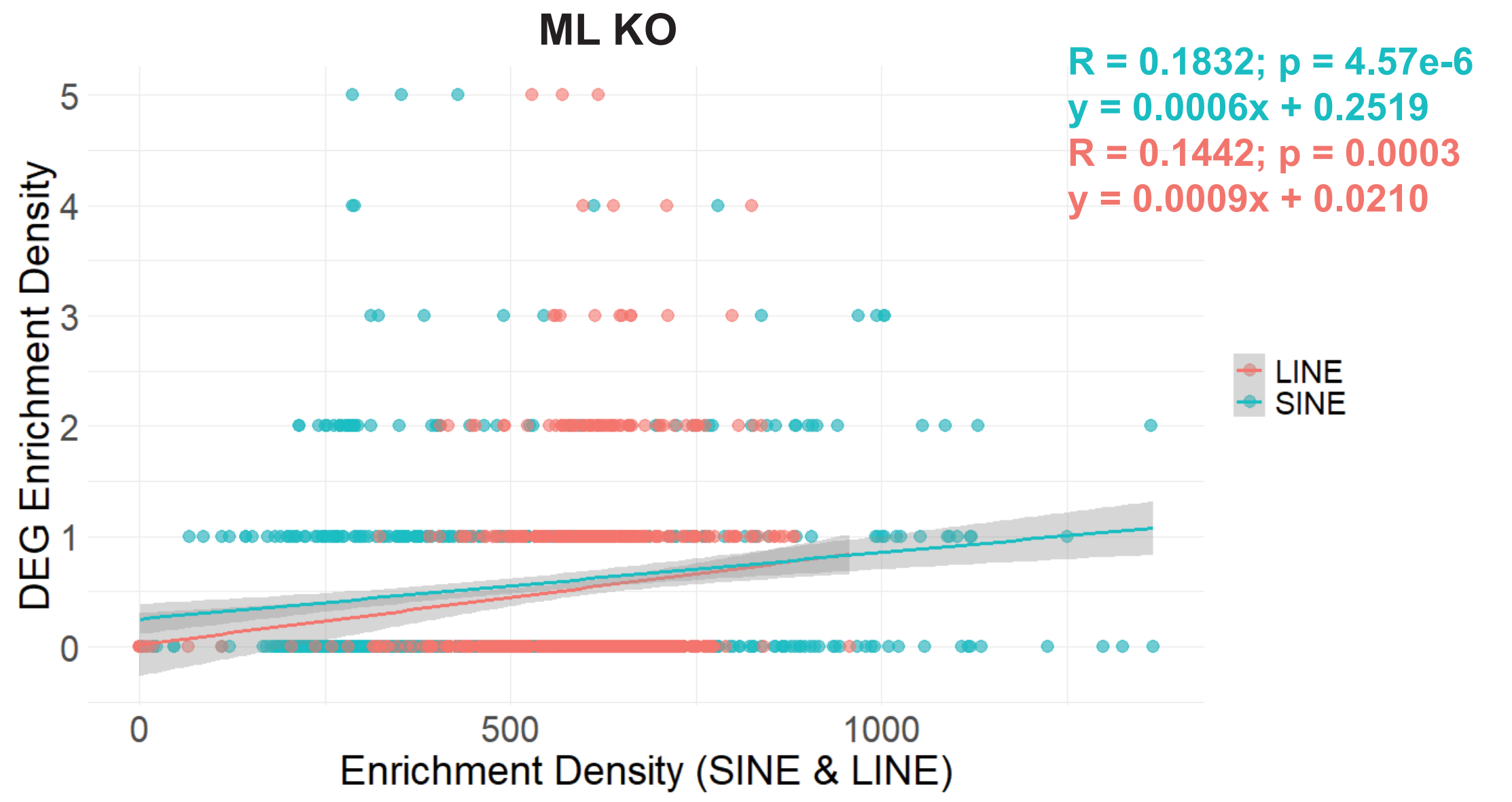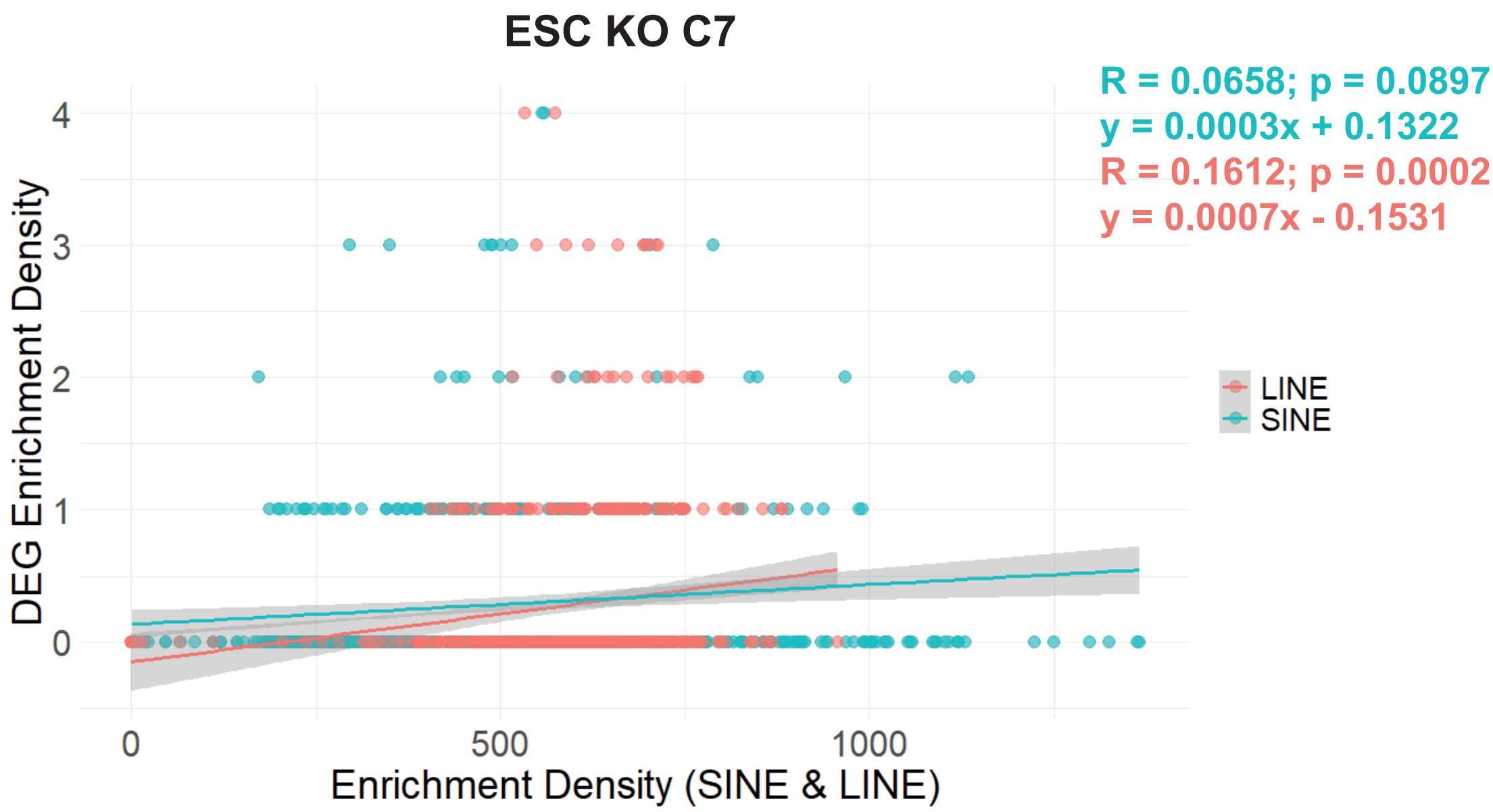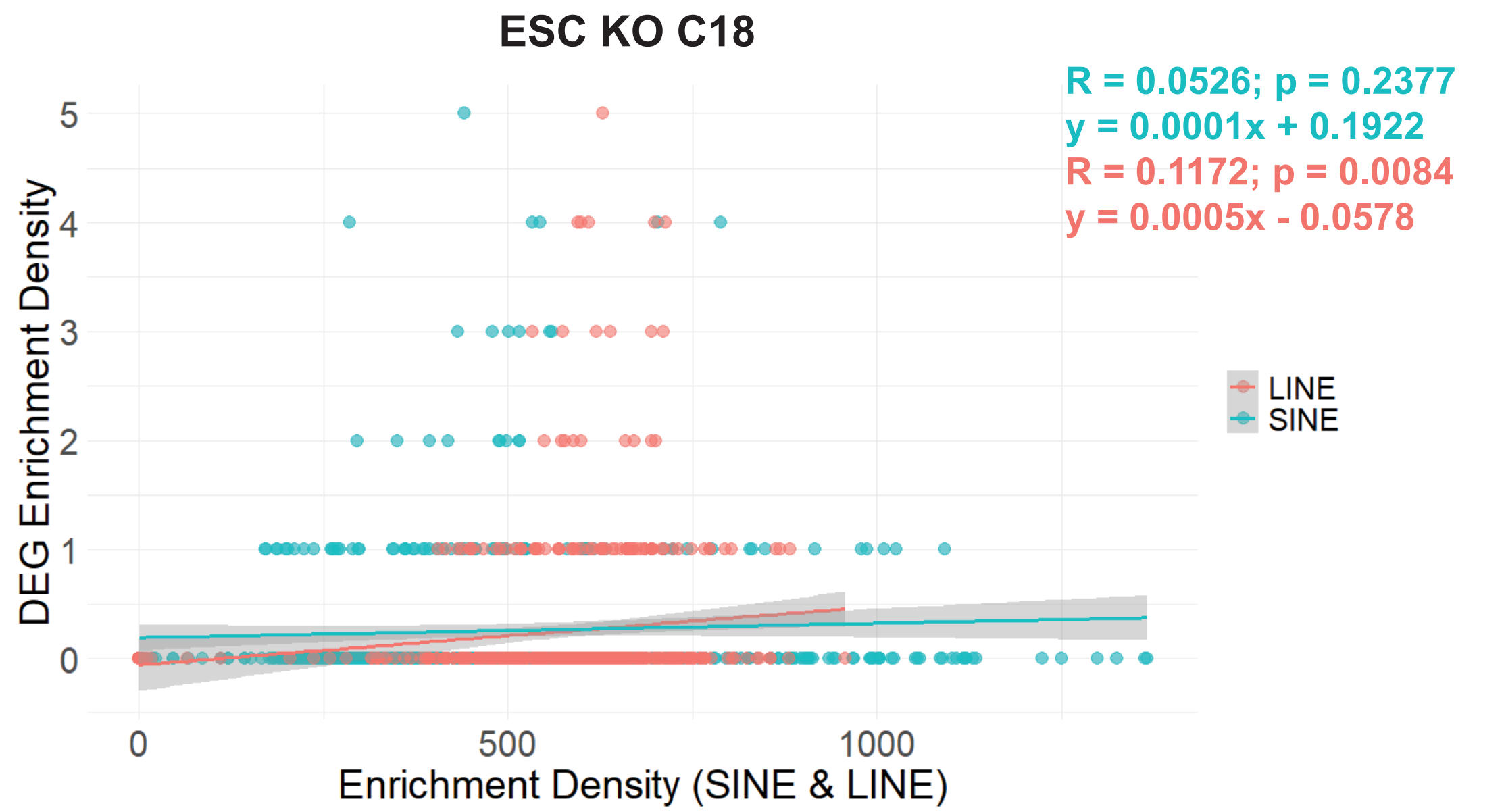

B

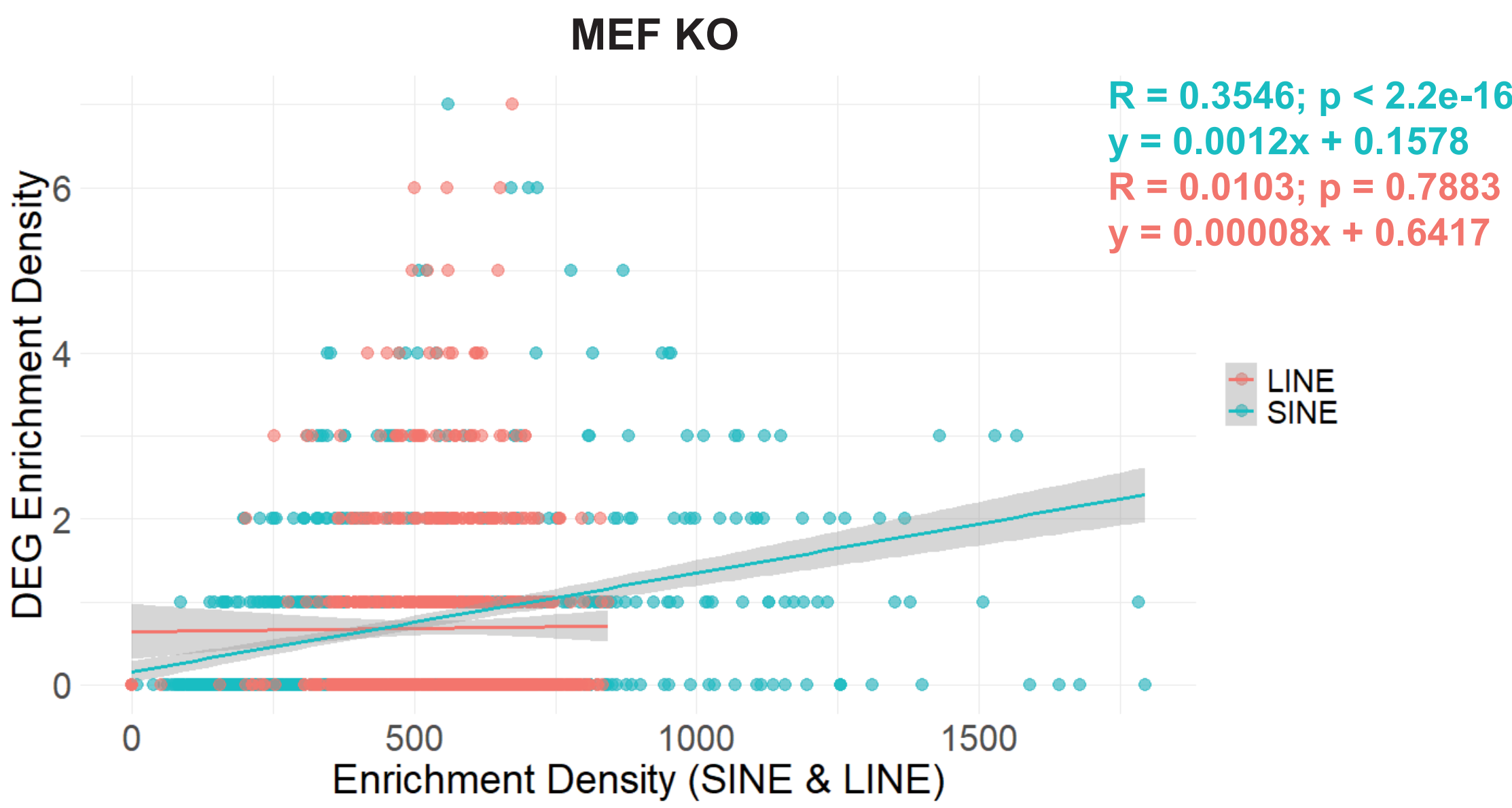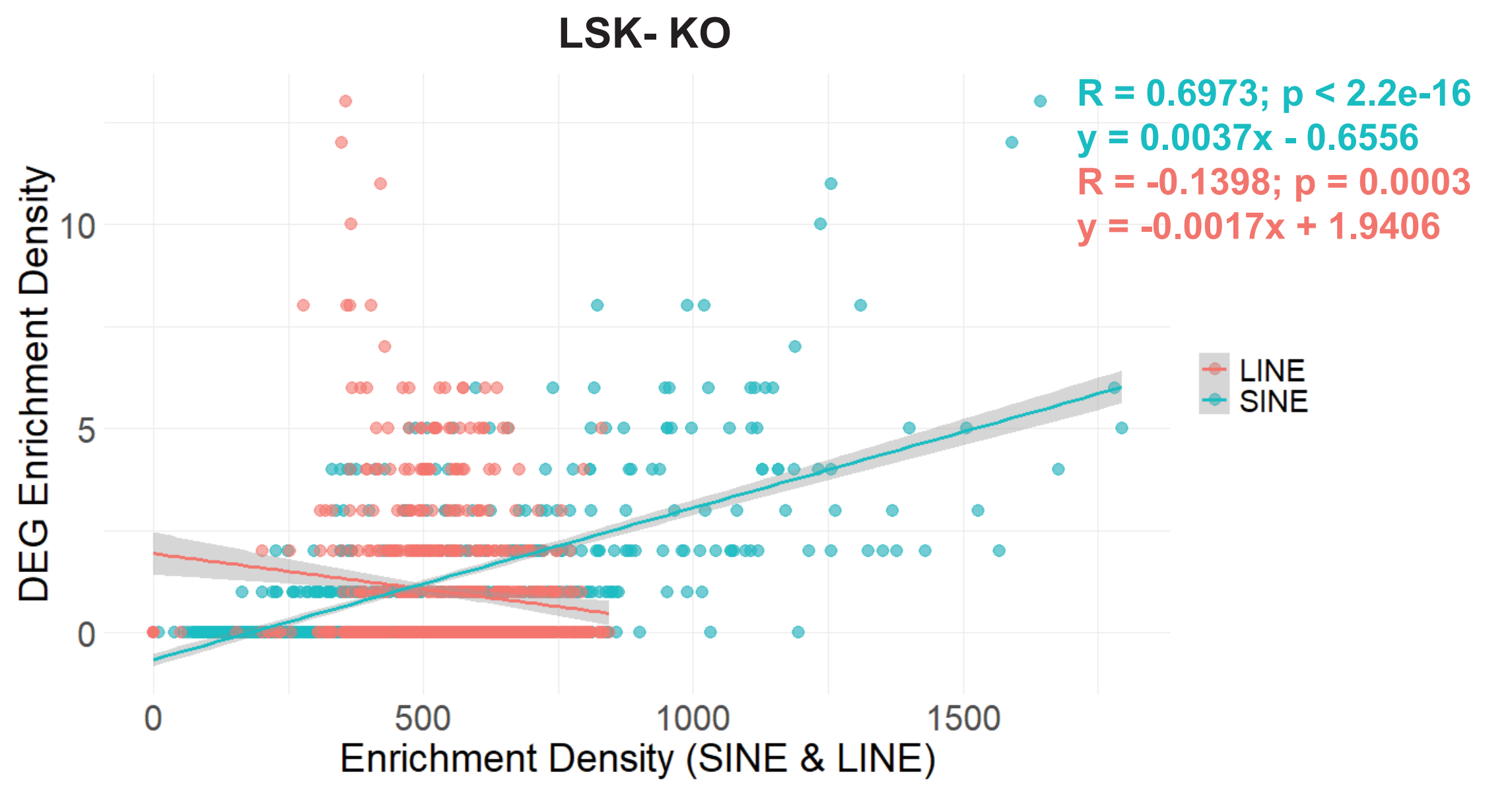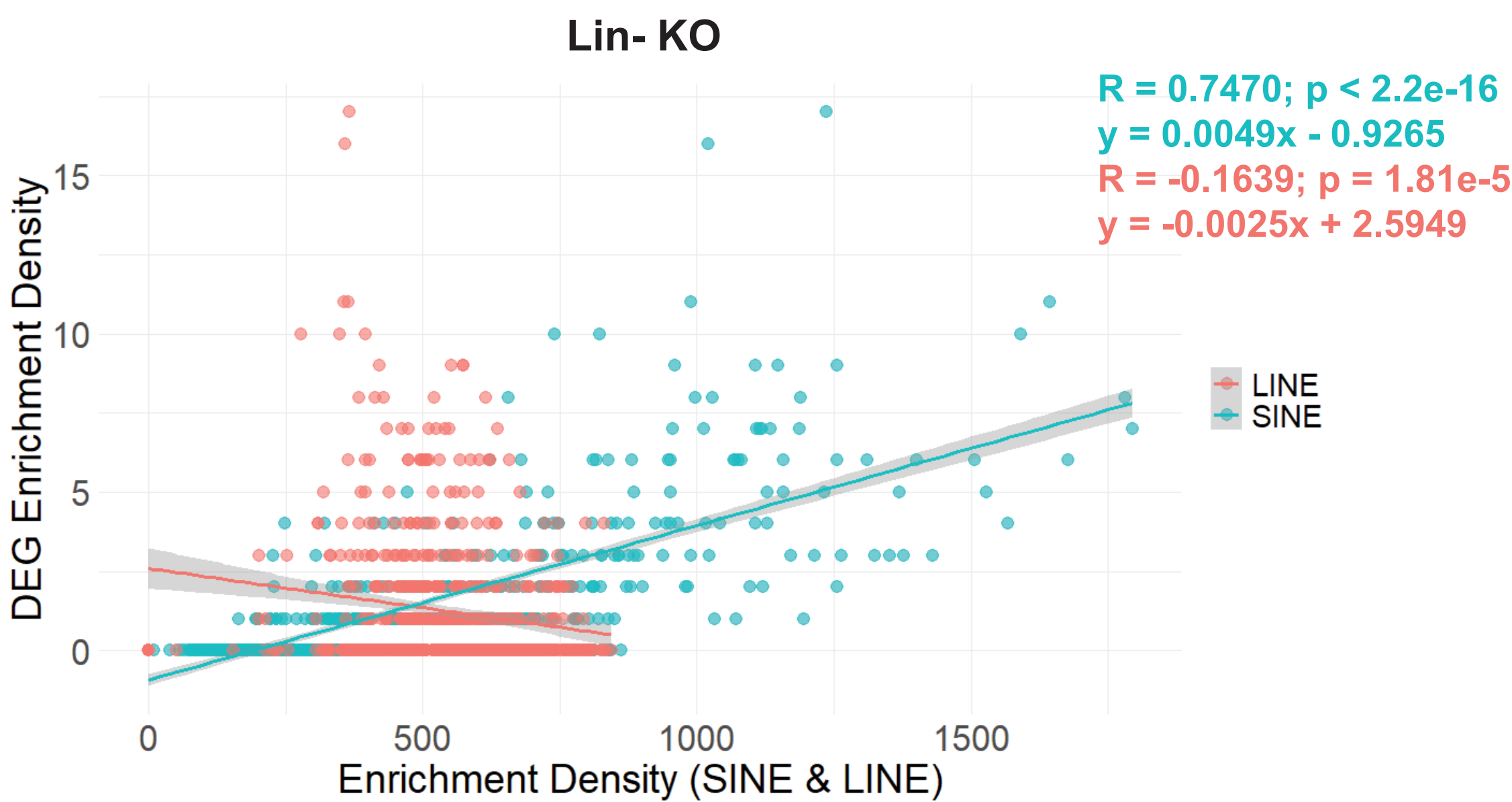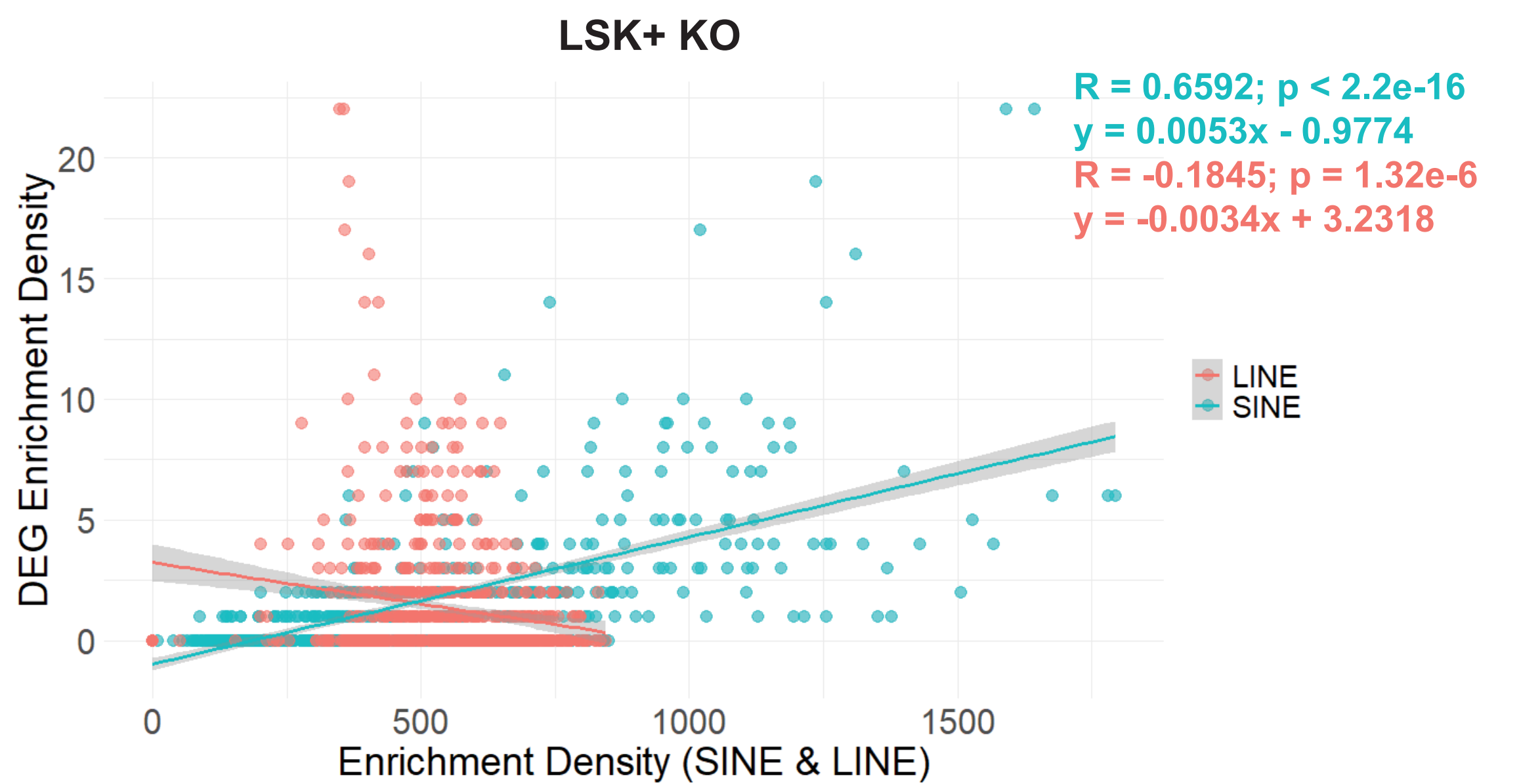

Supplementary Figure S3

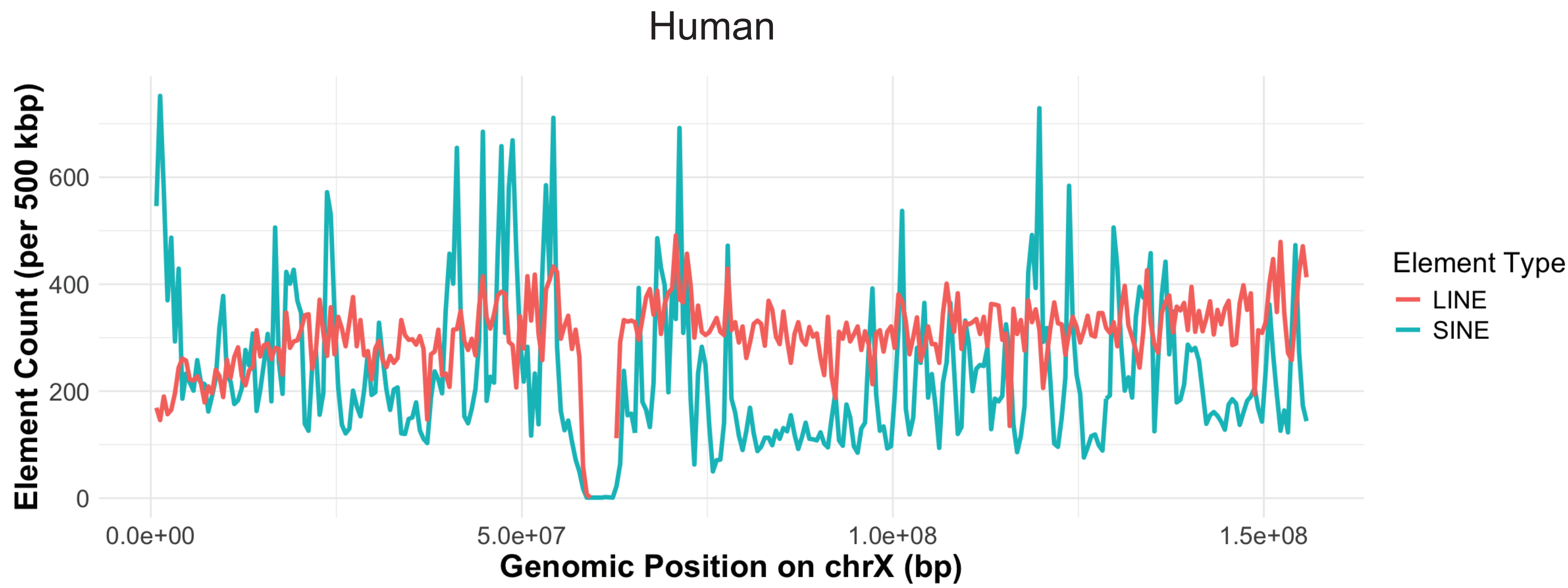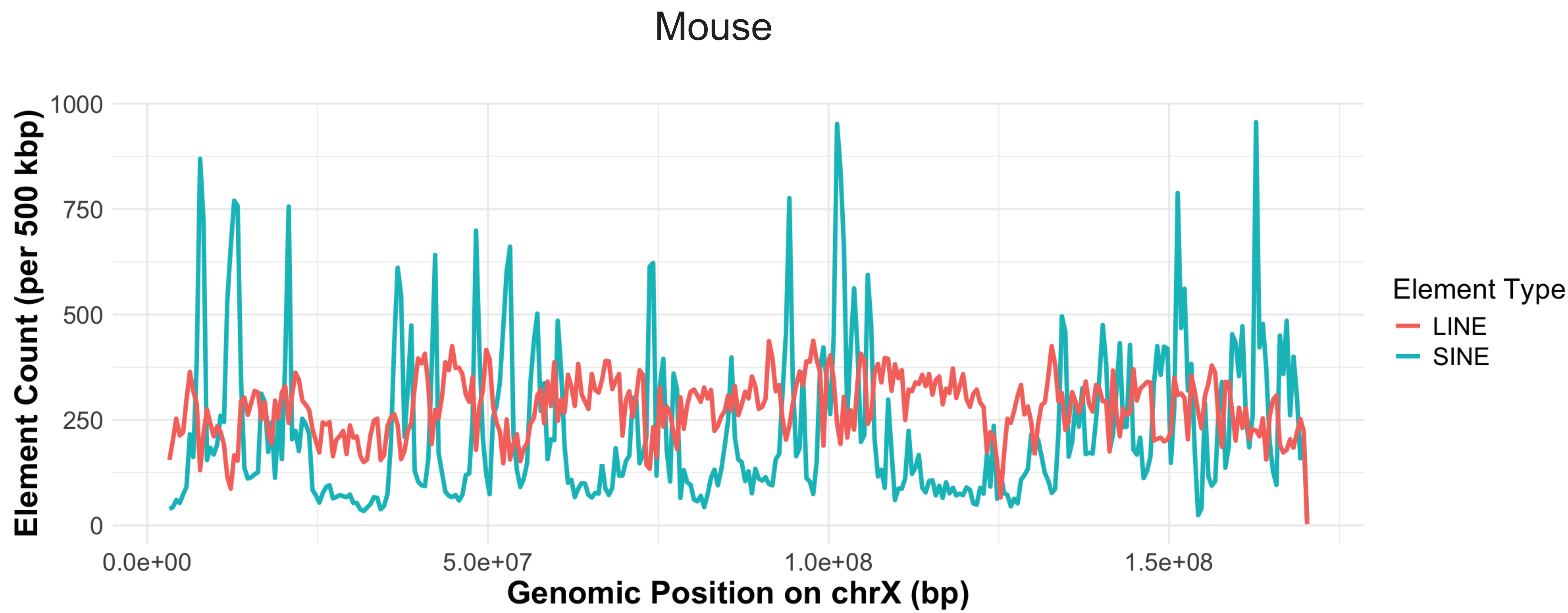

Supplement: Supplementary file 1 [file ncrna-11-00067-s001.zip › Supplementary/Supplementary Figures S1 to S3.pdf]
